# Essential oils for treating anxiety: a systematic review of randomized controlled trials and network meta-analysis

**DOI:** 10.3389/fpubh.2023.1144404

**Published:** 2023-06-01

**Authors:** Ling Tan, Fei-fei Liao, Lin-zi Long, Xiao-chang Ma, Yu-xuan Peng, Jie-ming Lu, Hua Qu, Chang-geng Fu

**Affiliations:** ^1^Xiyuan Hospital, China Academy of Chinese Medical Sciences, Beijing, China; ^2^Graduate School of Beijing University of Chinese Medicine, Beijing, China; ^3^National Cardiovascular Clinical Medical Research Center of TCM, Beijing, China

**Keywords:** anxiety, aromatherapy, essential oils, State-Trait Anxiety Inventory, network meta-analysis

## Abstract

**Background and purpose:**

The findings of clinical studies exploring essential oils (EOs) for anxiety remain disputed, and no studies have yet clarified the differences in the efficacy of EOs. The purpose of the study was to directly or indirectly compare the efficacy of different types of EOs on anxiety by pooling the results of randomized controlled trials (RCTs).

**Methods:**

PubMed, Cochrane Library, Embase, Scopus, Web of Science and the Cochrane Central Register of Controlled Trials (CENTRAL) databases were searched from inception to November 2022. Only full texts of RCTs that investigated the effects of EOs on anxiety were included. The trial data were extracted and the risk of bias was assessed by two reviewers independently. Pairwise meta-analysis and network meta-analysis were performed by Stata 15.1 or R 4.1.2 software.

**Results:**

Forty-four RCTs (fifty study arms) involving 10 kinds of EOs and 3419 anxiety patients (1815 patients in EOs group and 1604 patients in control group) were included. Pairwise meta-analyses showed that EOs were effective in reducing State Anxiety Inventory scores (SAIS) [WMD = −6.63, 95% CI−8.17, −5.08] and Trait Anxiety Inventory scores (TAIS) [WMD = −4.97, 95% CI−6.73, −3.20]. Additionally, EOs could decrease systolic blood pressure (SBP) [WMD = −6.83, (95% CI −10.53, −3.12), *P* < 0.001] and heart rate (HR) [WMD = −3.43, (95% CI −5.51, −1.36), *P* < 0.001]. Network meta-analyses demonstrated that regarding the outcome of SAIS, *Jasminum sambac (L.)Ait. (jasmine)* was the most effective with a weighted mean difference (WMD) of−13.61 (95% CrI−24.79, −2.48). Followed by *Citrus (citrus aurantium L.)*, which had a WMD of−9.62 (95% CrI−13.32, −5.93). Moderate effect sizes were observed for *Rosa rugosa Thunb*. (*damask rose)* (WMD = −6.78, 95% CrI−10.14, −3.49) and *Lavandula angustifolia Mill*. (*lavender)* (WMD = −5.41, 95% CrI−7.86, −2.98). Regarding the results of TAIS, *citrus aurantium L*. was the best ranked intervention with a WMD of−9.62 (95% CrI−15.62, −3.7). Moderate-to-large effect sizes were observed for *Citrus limon (L.) Burm. F*. (*lemon)* (WMD:−8.48; 95% CrI−16.67, −0.33) and *lavender* (WMD:−5.5; 95% CrI−8.7, −2.46).

**Conclusion:**

According to the comprehensive analysis, EOs are effective in reducing both state anxiety and trait anxiety, and *citrus aurantium L*. essential oil seems to be the most recommended type of EO for treating anxiety because of its significant effects in reducing SAIS and TAIS.

**Systematic review registration:**

https://www.crd.york.ac.uk/PROSPERO/, identifier: CRD42022331319.

## 1. Introduction

Anxiety disorders are one of the most disabling mental disorders and a major contributor to the global burden of disease (1). The prevalence of anxiety disorders is susceptible to political, social, economic and environmental changes, especially in the context of the era of the COVID-19 epidemic in the last 3 years, which has increased the prevalence of anxiety disorders by more than 25% (2, 3).

Currently, benzodiazepines (BDZs) and selective serotonin reuptake inhibitors (SSRIs) remain the cornerstones of treating anxiety disorders. These drugs provide a large short-term benefit, but their long-term efficacy is still limited and may cause certain side effects (4). In this context, complementary and alternative medicine (CAM) therapies are becoming increasingly accepted for their naturalness, affordability and fewer adverse effects. Aromatherapy, as its most important component, uses essential oils (EOs) to effectively balance the mind, body and spirit of the individual (5, 6). EOs are natural products from plants with small molecular weight and certain volatility (7). Essential oil (EO) molecules can affect the hypothalamus, autonomic nervous system and endocrine system (8), improve peripheral blood circulation, regulate blood pressure, pulse and respiration, and ultimately reduce anxiety (9–11).

Recently, a growing number of clinical trials have begun to explore the effects of EOs on anxiety due to various causes. However, the findings of EOs reduce anxiety remained somewhat controversial. Some studies have shown that EOs are effective in relieving anxiety, but others have concluded the opposite. Since the chemical constituents of EOs may vary greatly due to the species, origin place, extraction method, and concentrations in different clinical trials. Furthermore, different intervention procedures can also lead to differences in the effective constituents of which EOs exert their efficacy in the body (12). Therefore, the efficacy of EO may vary in different clinical trials even with the same type of EO. Herein, the efficacy of EOs in alleviating anxiety states still needs to be evaluated by meta-analysis. Of all the types of EOs, lavender is the most extensively studied. There is a meta-analysis has shown that *Lavandula angustifolia Mill*. (*lavender)* EO could ameliorate anxiety and its associated physiological parameters such as systolic blood pressure (SBP), diastolic blood pressure (DBP), heart rate (HR), and respiratory rate (RR) (13). Interestingly, another meta-analysis concluded that inhalation of *lavender* EO does not significantly reduce SBP (14). Furthermore, conventional pairwise meta-analyses are unable to integrate all the evidence from different types of EOs for anxiety at the same time, making it difficult to comprehensively and systematically evaluate the differences in the efficacy of various EOs and to select the best EO treatment regimen.

Given the limitations of the above studies, we used network meta-analysis combining both direct and indirect evidence to rank essential oils according to State Anxiety Inventory scores (SAIS) and Trait Anxiety Inventory scores (TAIS), and provide evidence-based medical evidence for the adoption of EOs for the treatment of anxiety disorders.

## 2. Methods

This study was conducted in accordance with the System Preferred Reporting Item Review and Meta-Analysis (PRISMA 2020) guidelines (15). The study was designed to explore the efficacies of common EOs on different causes of anxiety.

### 2.1. Protocol and registration

The protocol of the review was registered on the International Prospective Register of Systematic Reviews (PROSPERO) with registration number: CRD42022331319.

### 2.2. Data sources and search strategy

Two investigators (LT and F-FL) independently performed the database search. PubMed, Cochrane Library, Embase, Scopus, Web of Science and the Cochrane Central Register of Controlled Trials (CENTRAL) databases were searched with the terms combined medical subject headings (MeSH) and entry terms: [“oils, volatile”(MeSH) or “essential oils” or “volatile oils” or “aromatherapy” or “odorant”] AND [“anxiety” (MeSH) or “anxious” or “nervousness” or “hypervigilance” or “affect” or “mood” or “PHQ” or “GAD”] AND (“randomized controlled trial” or “clinical trial” or “RCT”) from inception to Nov 25, 2022. The detailed search strategies are listed in Supplementary Table S1. Moreover, the reference lists of the included studies and relevant review articles were manually checked to identify potential records that met the established criteria. Searches were not restricted by language.

### 2.3. Inclusion and exclusion criteria

The inclusion criteria were based on the Participant, Intervention, Comparison, Outcome, and Study design framework: (1) participant: adults (aged ≥ 18) with anxiety meet the diagnostic criteria of Diagnostic and Statistical Manual of Mental Disorders, fifth edition (DSM-V), or the International Statistical Classification of Diseases and Related Health Problems 10th Revision (ICD-10), or with scores of at least 20 in the Spielberger State-Trait Anxiety Inventory (STAI) questionnaire (mild anxiety), or have a specific trigger for anxiety and no olfactory problems and no allergies to aromatic substances; (2) intervention: inhalation of EOs (of any duration and frequency) in the trial group; (3) comparison: inhalation of unscented oil or only routine therapy in the control group; (4) outcomes: baseline and post-treatment SAIS, with or without TAIS and vital sign parameters, such as SBP, DBP, HR and RR; and (5) study design: randomized controlled trials (RCTs).

The following exclusion criteria were implemented: (1) the intervention was a mixture of EOs rather than a single type of EO; (2) studies with skewed baseline data for SAIS; (3) articles for which the full text was not available, and studies with incomplete primary outcome data; (4) duplicate publications; (5) studies were published as comments, conference abstracts, or letters to the editor.

### 2.4. Data extraction and quality assessment

After excluding trials that did not meet the eligibility criteria, two reviewers (LT and F-FL) independently read the full text of the remaining articles, and conducted data collection according to a validated extraction sheet based on the guidance of the Cochrane Handbook for Systematic Reviews of Interventions. The extracted data included: (1) general information (first author, publication year, and country); (2) participants (sample size, mean age, percentage of male participants, and causes of anxiety); (3) content of EOs (type of EO, cumulative duration of intervention, intervention doses); (4) details of the control group; and (5) outcomes (primary: SAIS and TAIS; secondary: SBP, DBP, HR, and RR). Disagreements were resolved through discussing with a third investigator (HQ).

Two reviewers independently assessed the risk of bias of the included studies with the Cochrane Risk of Bias assessment tool (RoB 2.0) (16). The overall risk of bias was classified as high risk (–), unclear risk (?), or low risk (+), based on the following domains: bias arising from the randomization process, bias due to deviations from intended interventions, bias due to missing outcome data, bias in measurement of the outcome, bias in selection of the reported result and overall risk of bias. An adapted version of the Grading of Recommendations Assessment, Development and Evaluation (GRADE) tool was used to evaluate the quality or confidence of evidence for each outcome by means of a web-based application Confidence in Network Meta-Analysis (CINeMA) (17).

### 2.5. Data synthesis and statistical analysis

A pair-wise meta-analysis was performed firstly by Der Simonian and Laird method. Then, a quantitative network meta-analysis of with random effects and uninformative priors based on Bayesian theorem was conducted to establish a comprehensive evaluation of the efficacy of EOs in treating anxiety.

The consistency model and the inconsistency model were applied to evaluate the hypothesis of overall consistency between networks in the meta-analysis. Then the deviance information criteria (DIC) of the two models were compared, and the model with the lower DIC value was selected. Subsequently, an ensemble node-split models were used to explore whether there was any statistical local inconsistency between direct and indirect comparisons (*P*-values > 0.1 indicated local consistency). A consistent model was accepted when there were no inconsistencies. Due to there were closed-loop structures between interventions, we conducted a loop inconsistency test to determine the existence of inconsistency according to the inconsistency factor (IF) value. If the IF value 95% CI contains 0, it indicates that there is no loop inconsistency.

Weighted mean difference (WMD) and 95% confidence interval (CI) were chosen as the effect sizes to report the results of the meta-analysis, while WMD and 95% credibility interval (CrI) as the effect sizes to report the results of the network meta-analysis. A WMD of 0.20 is considered a small difference between the trial and the control group; 0.50, a moderate difference; and 0.80, a large difference. The Higgins I-squared (I^2^) index was used to estimate the potential heterogeneity. Random effects models were chosen to compare treatment efficacy considering the clinical and methodological heterogeneity between studies. Each model was calculated by generating 10, 000 sample iterations, with an initial burn-in period of 2, 000 iterations (thin = 1). To rank the interventions, the probability of each intervention being ranked first, second, etc was calculated. The efficacy of each intervention was also ranked by calculating the surface under the cumulative ranking curve (SUCRA).

In view of the significant heterogeneity, exploratory subgroup analyses were conducted based on type of EO, country, cause of anxiety, and accumulative duration of intervention for SAIS, TAIS, SBP, DBP, HR, and RR. Meta-regression analyses were performed based on the accumulative duration of intervention, baseline SAIS and baseline TAIS. Additionally, sensitivity analyses were performed to assess the robustness of the results based on excluding the studies with < 30 patients per intervention arm to ensure that large or overestimated treatment benefits were excluded, since small studies tend to have larger effects compared with larger studies. Potential publication bias was evaluated by visual assessment of funnel plots.

Conventional pairwise meta-analyses were performed by STATA version 15.1 (Stata Corp, College Station, TX, USA). Network meta-analyses were conducted using R version 4.1.2 software (“netmeta” and “gemtc” 1.0.1 packages). Network pictures were created to visualize network geometry and node connectivity.

## 3. Results

### 3.1. Study selection

**Figure 1:**
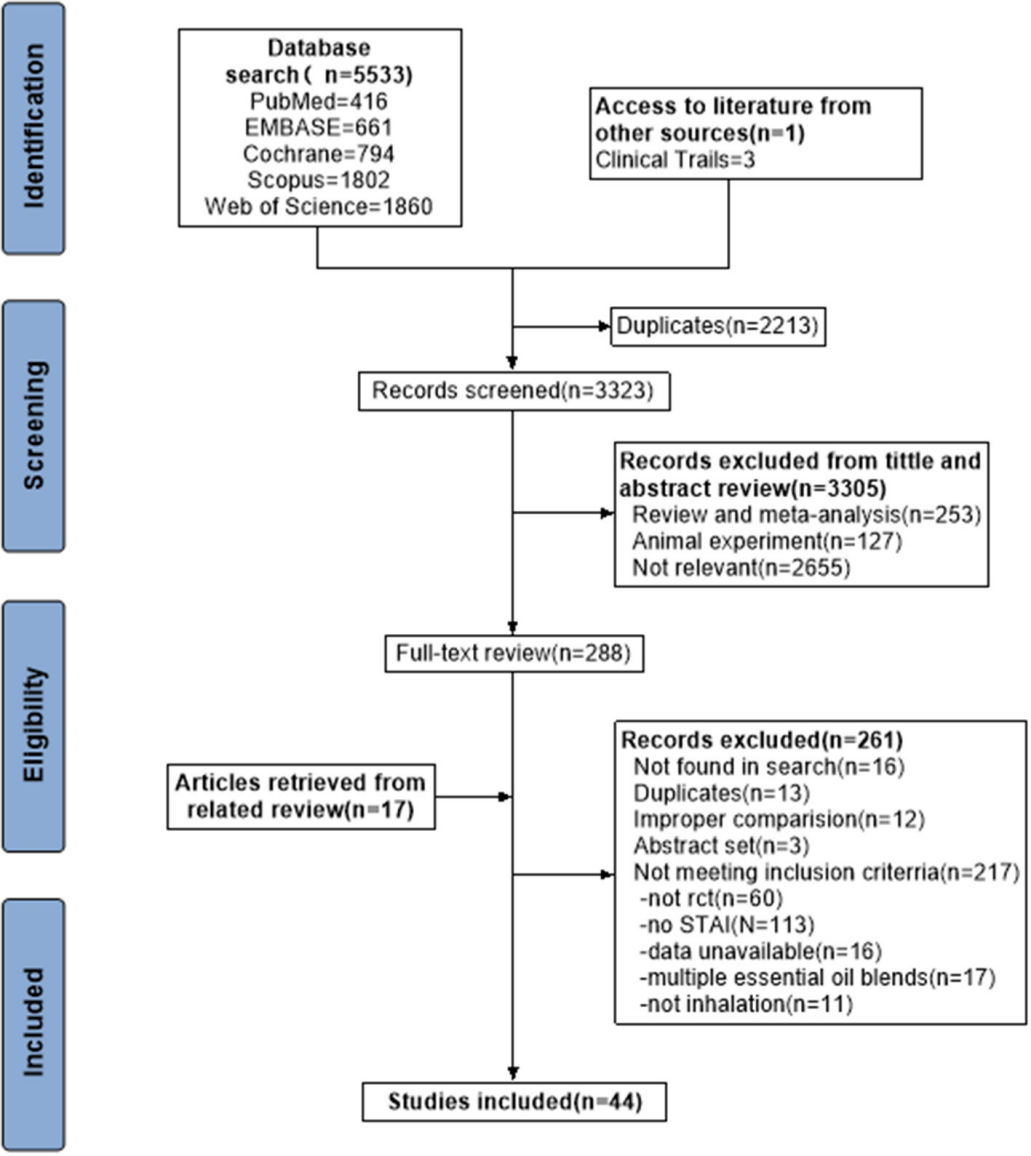
Flow diagram of study identification, screening, eligibility assessment, and inclusion.

The screening process of a PRISMA study flow diagram was shown in Figure 1. We retrieved 5, 553 articles through a systematic search, of which 5, 265 were excluded after a preliminary screening of titles and abstracts since they were completely unrelated to the topic of “essential oil for anxiety”. Two hundred and eighty-eight articles were potentially eligible records whose full text was reviewed. Finally, 44 articles were included in present review (14, 18–60).

### 3.2. Characteristics of included trials

Publication dates ranged from 2010 to 2022 (median, 2016), with 86% of trials published after 2016. Most trials were conducted by Iranian scholars (33/44, 75% trials). The second highest number of trials was conducted in Turkey, with eight. The EOs involved *lavender, Rosa rugosa Thunb*. (*damask rose), Citrus (citrus aurantium L.), Phyla nodiflora (Linn.) E. L. Greene (lippia alba), Mentha haplocalyx Briq. (mint), Citrus limon (L.) Burm. F*. (*lemon), Eucalyptus citriodora Hook. f. (lippia citriodora), Pelargonium hortorum Bailey (geranium), Jasminum sambac (L.)Ait. (jasmine)*, and *Balsam capivi, jesuits'resin (copaiba)*. Fourteen trials explored the effects of EOs on operation-related anxiety, while 12 trials about the invasive examinations induced anxiety. Overall, 44 studies involving 3,419 anxiety patients (1,815 patients in EOs group and 1604 patients in control group) were included (14, 18–60). The detailed characteristics of the studies included in this review were listed in Table 1.

**Table 1:** Characteristics of included studies.

**Author, year**	**Country**	**Participants (EG, CG)**		**Causes of anxiety**	**Content of intervention (EG, CG)**			**Scale**	**Physiological parameters**
		**Sample size (male/female)**	**Mean age (M** ±**SD or range)**		**Essential oil type**	**Duration of intervention**	**Volume of essential oil**		
Abbasijahromi et al. (14)	Iran	EG1:30 EG2:30 CG:30	EG1:29.73 ± 5.29 EG2:26.79 ± 5.53 CG:27.6 ± 5.31	C-section	EG1: Lavender EG2: Damask rose	30 min	3 drops	STAI	NR
Alvarado-García et al. (18)	Perú	EG1:27 (12/15) EG2:27 (12/15) CG:26 (12/14)	EG1:39.50 ± 5.97 EG2:39.84 ± 5.90 CG:39.87 ± 5.85	Spontaneous anxiety	EG1: Lippia alba EG2: Lippia citriodora	30 min	2 drops	STAI	NR
Amzajerdi et al. (19)	Iran	EG:33 (0/33) CG:33 (0/33)	EG:26.97 ± 4.57 CG:28.4 ± 3.91	Pregnant	Mint	280 min	4 drops	SAI	NR
Babatabar Darzi et al. (20)	Iran	EG1:40(25/15) EG2:40(28/12) CG:40 (19/21)	EG1:60.50 ± 5.26 EG2:58.05 ± 5.26 CG:62.27 ± 6.49	Open Heart Surgery	EG1: Damask rose EG2: Lavender	15 min	3 drops	SAI	NA
Bahadori et al. (21)	Iran	EG:30 (12/18) CG:30 (10/20)	EG:31.23 ± 4.41 CG:33.2 ± 7.49	Operating room nurses	Damask Rose	10 min	2 drops	SAI	NR
Bakhsha et al. (22)	Iran	EG:50 CG:50	NR	Preoperative Anxiety	Lavender	1 min	NR	SAI	NR
Beyliklioglu and Arslan (23)	Turkey	EG:40 CG:40	EG:51.48 ± 17.31 CG:48.00 ± 10.63	Before Breast Surgery	Lavender	20 min	3.5 drops	SAI	NR
Eslami et al. (24)	Iran	EG:30 (15/15) CG:30 (15/15)	EG:51.93 ± 7.26 CG:51.47 ± 5.87	Candidates for surgery	*Citrus aurantium* L.	20 min	2 drops	STAI	NR
Eslami et al. (25)	Iran	EG1:30 (15/15) EG2:30 (15/15) CG:30 (15/15)	EG1:51.87 ± 7.81 EG2:51.93 ± 7.26 CG:51.47 ± 5.87	Laparoscopic cholecystectomy	EG1: Lavender EG2: *Citrus aurantium* L.	20 min	2 drops	STAI	NR
Farzaneh et al. (26)	Iran	EG:19 CG:19	EG:49.21 ± 10.63 CG:47.74 ± 15.47	Preoperative Anxiety	Damas Rose	10 min	3 drops	STAI	NR
Fayazi et al. (27)	Iran	EG:36 CG:36	NR	Preoperative Anxiety	Lavender	20 min	2 drops	SAI	NA
Ganji et al. (28)	Iran	EG:44 (32/12) CG:44 (27/17)	EG:46.0 ± 11.5 CG:44.0 ± 13.5	Kidney stones	Damask Rose	15 min	3 drops	SAI	NR
Haddadi et al. (29)	Iran	EG:40 (15/25) CG:40 (17/23)	NR	Myaocardial infarction	Damask Rose	225 min	3 drops	SAI	NR
Hamdamian et al. (30)	Iran	EG:55 CG:55	EG:25.87 ± 5.17 CG:26.24 ± 5.15	During first stage of labor	Damask Rose	30 min	2 drops	SAI	NR
Hekmatpou et al. (31)	Iran	EG:30 CG:30	NR	Fractured limbs admitted	*Citrus aurantium* L.	360 min	4 drops	SAI	NR
Hu et al. (32)	China	EG:14 (9/5) CG:13 (6/7)	NR	Colonoscopy-related surgery	*Citrus aurantium* L.	5 min	1 drop	SAI	SBP, DBP, RR, HR
Jirdehi et al. (33)	Iran	EG1:35 (15/20) EG2:35 (11/24) CG:35 (19/16)	NR	Candidate for endoscopy	EG1: Lavender EG2: Damask Rose	30 min	2 drops	SAI	NR
Jodaki et al. (34)	Iran	EG:30 (16/14) CG:30 (15/15)	EG:62.8 ± 11.8 CG:61.5 ± 12.75	Cardiac disease	Damask Rose	1440 min	5 drops	SAI	NR
Jokar. et al. (35)	Iran	EG:31 (0/31) CG:31 (0/31)	EG:55.95 ± 5.70 CG:53.56 ± 2.67	Perimenopause	Lavender	560 min	2 drops	STAI	NR
Kasar et al. (36)	Turkey	EG:22 (6/16) CG:22 (3/19)	EG:48.6 ± 12.0 CG:48.1 ± 11.9	Trigger Point Injection	Lavender	12.5 min	5 drops	SAI	NR
Mokhtari et al. (37)	Iran	EG:30 (16/14) CG:30 (15/15)	NR	Burn	Damask Rose	480 min	5 drops	SAI	NR
Moradi et al. (38)	Iran	EG:40 (21/19) CG:40 (24/16)	EG:55.71 ± 1.65 CG:55.95 ± 1.76	Coronary arteriography	*Citrus aurantium* L.	17.5 min	4 mL	SAI	SBP, DBP, RR, HR
Moslemi et al. (39)	Iran	EG:70 (29/41) CG:70 (37/33)	EG:56.76 ± 11.39 CG:56.69 ± 11.37	Acute coronary syndrome	*Citrus aurantium* L.	20 min	1.5 drops	SAI	NR
Ozkaraman et al. (40)	Turkey	EG:30 (6/24) CG:20 (3/17)	EG:57.73 ± 12.81 CG:57.55 ± 12.87	Chemotherapy	Lavender	150 min	3 drops	STAI	NR
Pasyar et al. (41)	Iran	EG:30 (9/21) CG:30 (10/20)	EG:38.10 ± 12.37 CG:38.4 ± 9.58	Laparoscopic cholecystectomy	*Citrus aurantium* L.	20 min	2 drops	SAI	NR
Pimenta et al. (42)	Brazil	EG:14 CG:14	NR	Chronic Myeloid Leukemia	*Citrus aurantium* L.	30 min	10 mL	SAI	SBP, DBP, RR, HR
Rambod et al. (43)	Iran	EG:50 (27/23) CG:50 (28/22)	EG:61.42 ± 14.98 CG:61.84 ± 11.3	Acute myocardial infarction	Lemon	2160 min	5 drops	STAI	SBP, DBP, HR
Reyes et al. (44)	Philippines	EG:25 (14/11) CG:25 (13/12)	EG:52.04 ± 10.38 CG:57.52 ± 13.51	Puncture for hemodialysis patients	*Citrus aurantium* L.	5 min	3 drops	SAI	NR
Sahin et al. (45)	Turkey	EG:36 (19/17) CG:38 (23/15)	EG:50.75 ± 18.02 CG:53.62 ± 11.03	Puncture for hemodialysis patients	Lavender	15 min	5 drops	STAI	NR
Saritas et al. (46)	Turkey	EG:45 (30/15) CG:45 (26/19)	EG:49.26 ± 14.57 CG:50.62 ± 14.58	ERCP	Lavender	30 min	4 drops	SAI	SBP, DBP, HR
Shirzad et al. (47)	Iran	EG:34 (12/22) CG:34 (14/20)	EG:28.2 ± 7 CG:27.1 ± 5.9	Septorhinoplasy and Rhinoplasty	Lavender	20 min	3 drops	STAI	NA
Soleimani et al. (48)	Iran	EG:32 (16/16) CG:32 (16/16)	NR	acute coronary syndrome	Mint	60 min	3 drops	SAI	NA
Soto-Vásquez et al. (49)	Peru	EG:28 (12/16) CG:27 (13/14)	NR	Spontaneous anxiety	Lippia alba	360 min	4 drops	STAI	NR
Stanley et al. (50)	Singapore	EG:39 (17/22) CG:36 (16/20)	EG:61.6 ± 7.0 CG:63.25 ± 7.7	Cataract Surgery	Lavender	20 min	20 drops	SAI	SBP, DBP, RR, HR, SpO2
Tahmasbi et al. (51)	Iran	EG:45 (18/27) CG:46 (23/23)	NR	Coronary angiography	Lavender	3 min	2 drops	STAI	NA
Tahmasebi et al. (52)	Iran	EG1:33 (12/21) EG2:35 (18/17) CG:33 (14/19)	EG1:60.12 ± 6.79 EG2:57.80 ± 6.21 CG:58.64 ± 6.11	Coronary angiography	EG1: Lavender EG2: *Citrus aurantium* L.	20 min	2 drops	SAI	SBP, DBP, RR, HR, SpO2
Wen et al. (53)	China	EG:50 (17/33) CG:50 (14/36)	EG:47.2 ± 14.5 CG:47.1 ± 14.2	MRI examinations	Lavender	20 min	8 drops	SAI	NR
Fakarian and Tabatabaeichehr (54)	Iran	EG:49 (24/25) CG:48 (21/27)	EG:23 ± 7 CG:21 ± 5	The first stage of labor	Geranium	20 min	NR	SAI	SBP, DBP, RR, HR
Babaii et al. (55)	Iran	EG:30 CG:30	EG:53.63 ± 9.99 CG:56.96 ± 7.89	Cardiac Catheterization	Damask Rose	18 min	NR	STAI	NR
Inci and Çetinkaya (56)	Turkey	EG:48 (30/18) CG:48 (28/20)	EG:60.89 ± 8.74 CG:56.54 ± 11.62	Coronary angiography	Lavender	15 min	5 drops	SAI	SBP, DBP, RR, HR
Karan (57)	Turkey	EG:63 (13/50) CG:63 (17/46)	NR	Oral surgery	Lavender	3 min	NR	SAI	SBP, DBP, RR, HR
Yadegari et al. (58)	Iran	EG:42 (33/9) CG:42 (33/9)	EG:35.55 ± 12.75 CG:36.26 ± 13.39	Laparotomy	Jasmine	60 min	2 drops	SAI	NR
Zhang et al. (59)	China	EG:11 CG:11	NR	Mental Workload	Copaiba	20 min	NR	STAI	NA
Tugut et al. (60)	Turkey	EG:78 CG:78	EG:35.0 ± 9.7 CG:33.5 ± 12.4	Gynecological examination	Lavender	12.5 min	NR	SAI	NR

EG, Experimental group (When there are two controlled trials in the same literature, EG1 and EG2 are used to represent the two experimental groups respectively); CG, Control group; NR, Not Reported; NA, No Analyzed (The study reported, but did not present original data that could be analyzed); STAI, Spielberger State-Trait Anxiety Inventory; SAI, State Anxiety Inventory.

### 3.3. Assessment of risk of bias

**Figure 2:**
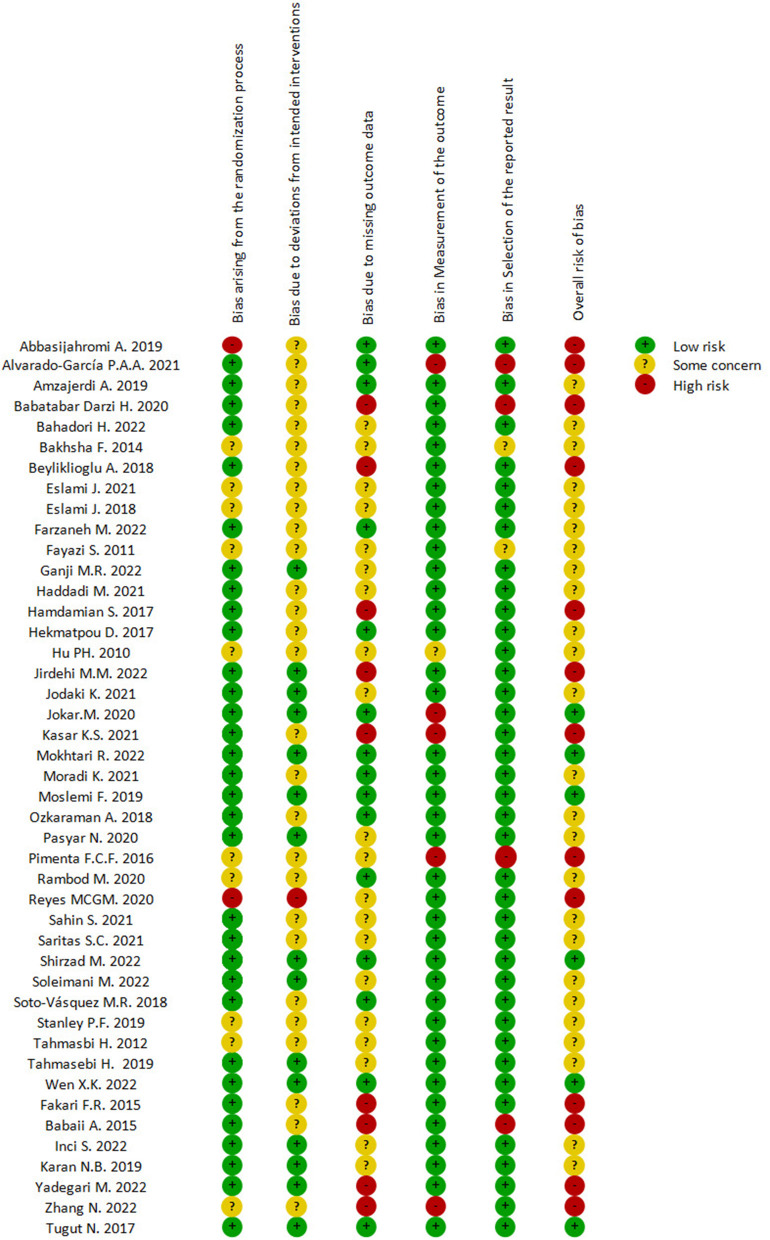
Summary of the risk of bias assessment.

**Figure 3:**
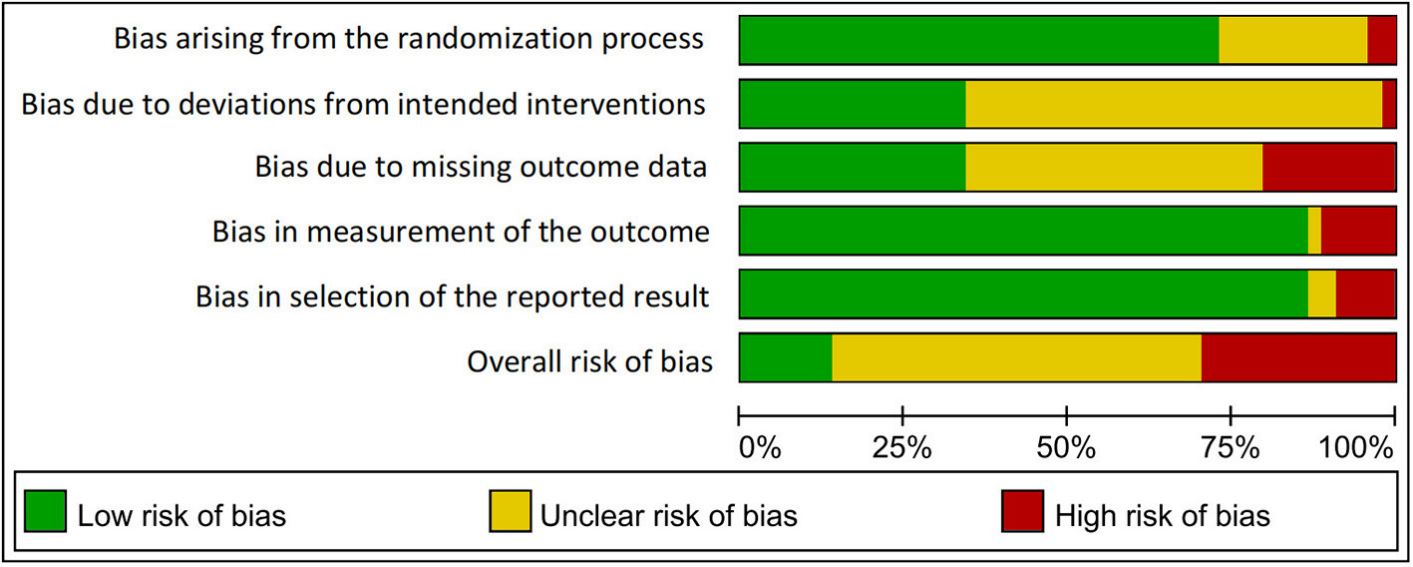
Risk of bias graph.

As shown in Figures 2, 3, all included trials were assessed for risk of bias (RoB 2.0) tool. Seventeen studies had a high risk of bias (18, 20, 22, 23, 30, 33, 35, 37, 38, 42–44, 46, 54, 55, 58, 59), and one-quarter of the studies (25%) were at “unclear risk of bias” (24, 25, 27, 29, 31, 32, 36, 45, 48, 50, 51), mostly due to deviations from the intended interventions and selection of the reported results. The remaining 16 studies were at low risk of bias (14, 19, 21, 26, 28, 34, 39–41, 47, 49, 52, 53, 56, 57, 60), with all assessed domains in these studies being at low risk.

### 3.4. Direct pairwise meta-analysis

#### 3.4.1. State anxiety inventory

**Figure 4:**
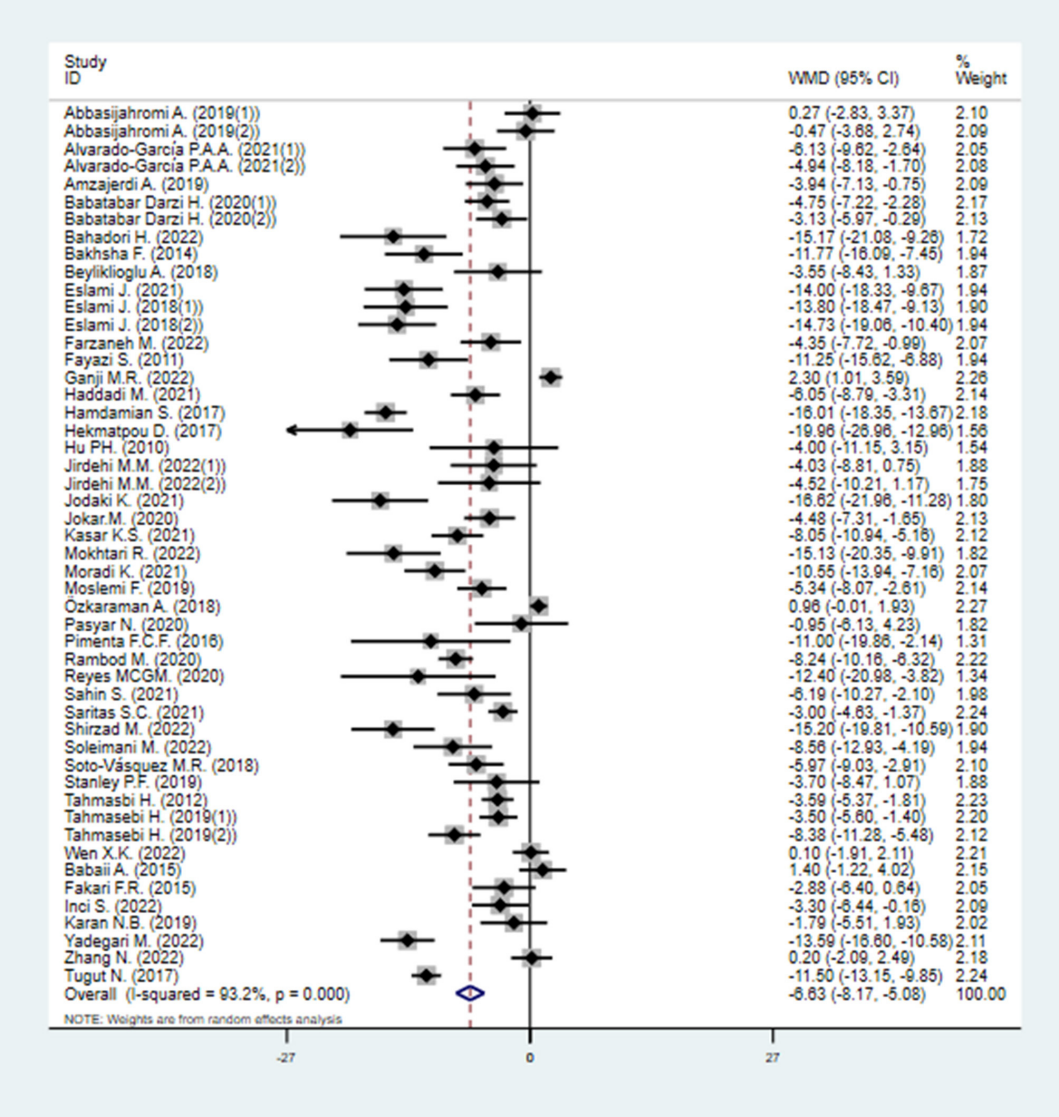
Direct pairwise random-effects meta-analyses of SAIS. SAIS, State Anxiety Inventory scores.

Forty-four trials (50 study arms), including 3,419 anxiety patients (1,815 patients in EOs group vs. 1,604 patients in control group), evaluated the effects of EOs on their anxiety by State Anxiety Inventory (SAI). The pooled WMD showed that EOs therapy led to a significant lower level of state anxiety compared to control group [WMD = −6.63 (95% CI −8.17, −5.08), *P* < 0.001], with moderate heterogeneity (*I*^2^ = 93.2%, *P* < 0.001) (Figure 4).

#### 3.4.2. Trait anxiety inventory

**Figure 5:**
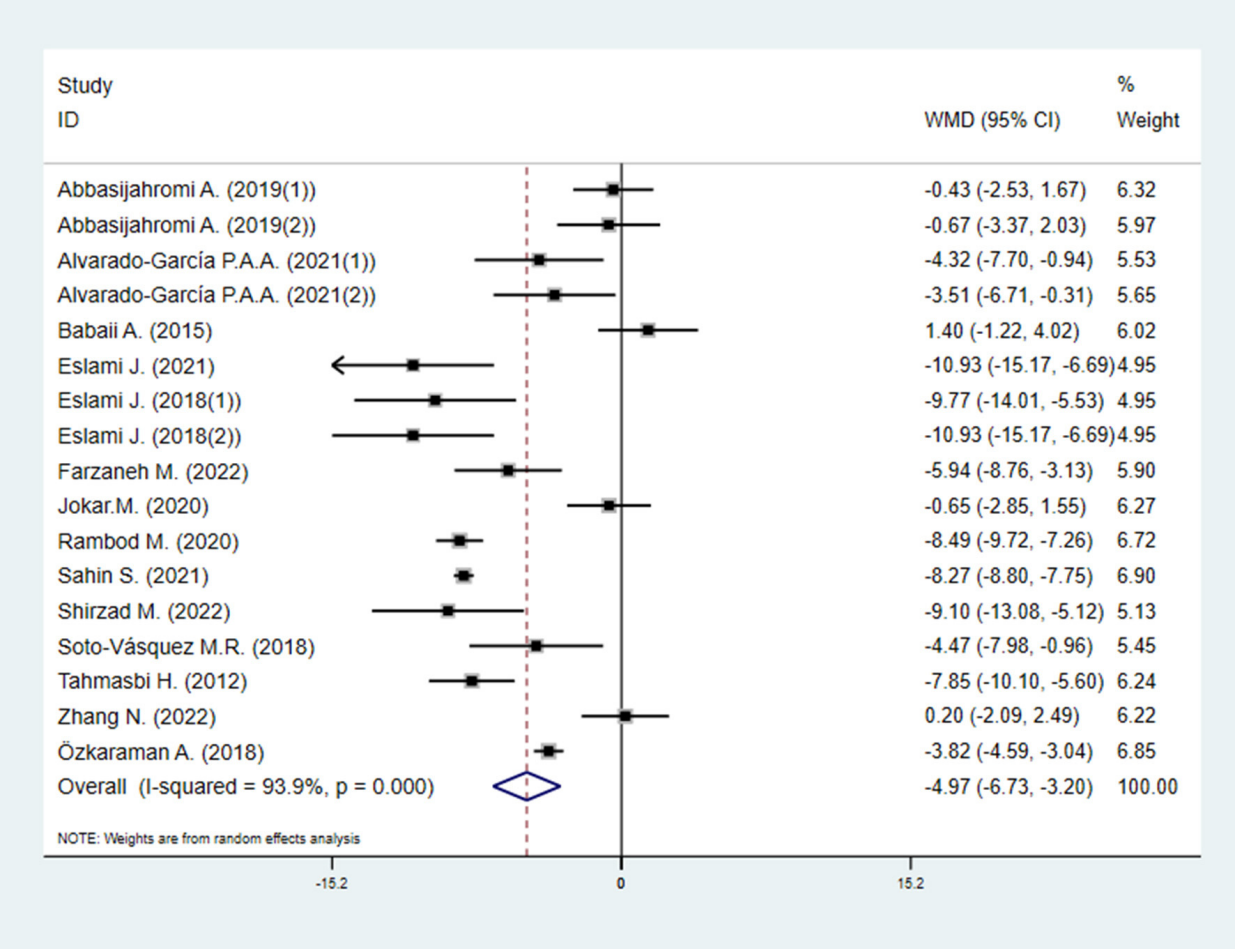
Direct pairwise random-effects meta-analyses of TAIS. TAIS, Trait Anxiety Inventory scores.

A total of 14 studies (17 study arms), including 940 anxiety patients (518 patients in EOs group vs. 422 patients in control group), investigated the effects of EOs therapy on anxiety by Trait Anxiety Inventory (TAI). Pooled effect sizes from the eligible studies indicated that EOs therapy significant lowered the level of trait anxiety compared to control group [WMD = −4.97 (95% CI −6.73, −3.20), *I*^2^ = 93.9%, *P* < 0.001] (Figure 5).

#### 3.4.3. Vital signs

**Figure 6:**
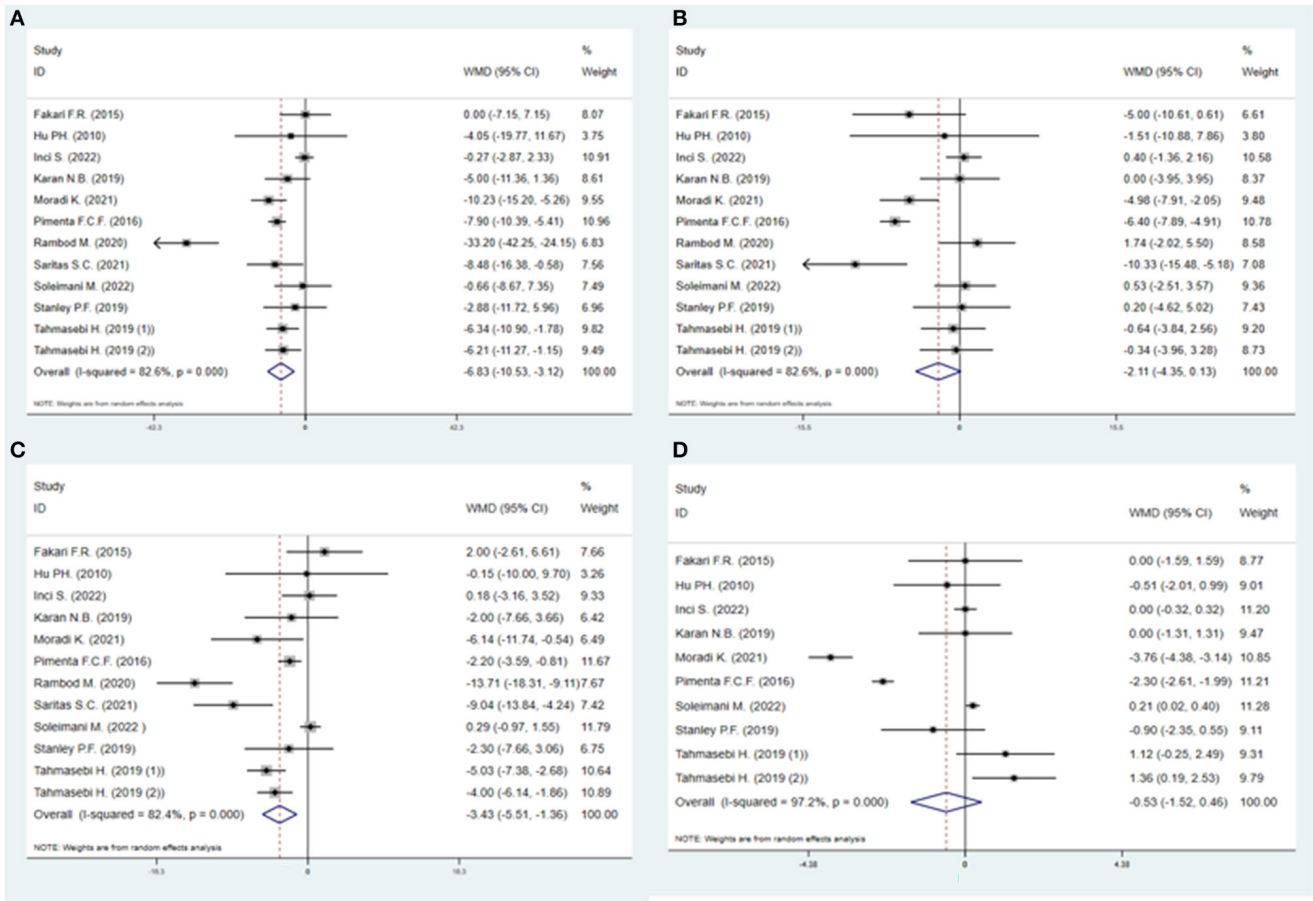
Direct pairwise random-effects meta-analyses of vital signs. **(A)** Systolic blood pressure (SBP); **(B)** Diastolic blood pressure (DBP); **(C)** Heart rate (HR); **(D)** Respiratory rate (RR).

As presented in Figure 6, compared with the control, EOs could decrease SBP [WMD = −6.83 (95% CI−10.53, −3.12), *P* < 0.001] (Figure 6A) and HR [WMD = −3.43 (95% CI−5.51, −1.36), *P* < 0.001] (Figure 6C). The effects of EOs on DBP and RR were also evaluated. EOs had a tendency of decreasing DBP [WMD = −2.11 (95% CI−4.35, 0.13), *P* < 0.001] (Figure 6B) and RR [WMD = −0.53 (95% CI−1.52, 0.46), *P* < 0.001] (Figure 6D), but they were not statistically significant.

### 3.5. Network meta-analysis

#### 3.5.1. Indirect-comparisons meta-analysis for EOs on state anxiety scores

**Figure 7:**
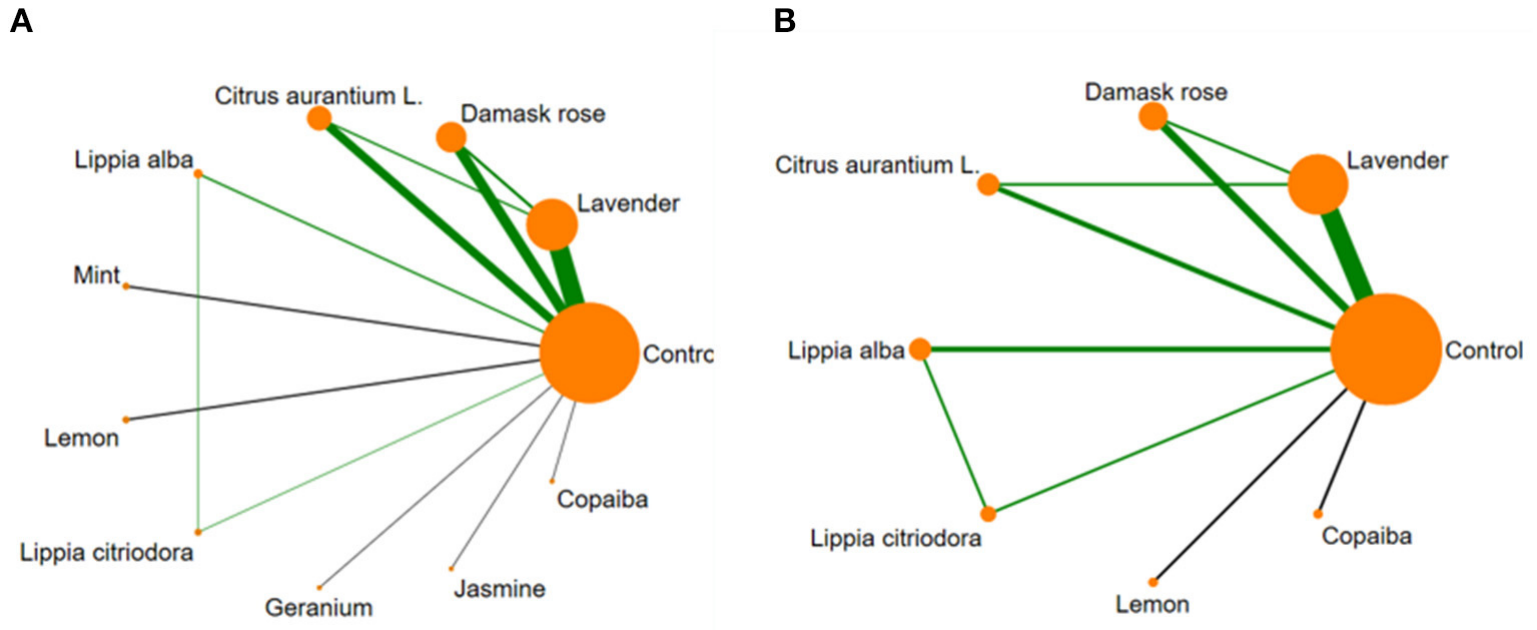
Network meta-analysis of available comparisons between 10 essential oils and the control. **(A)** State Anxiety Inventory scores (SAIS); **(B)** Trait Anxiety Inventory scores (TAIS). Line width is proportional to the number of trials that included each pair of treatments (direct comparisons). Circle size is proportional to the total number of participants for each treatment in the network.

The network consisted of 38 studies with two arms and 6 studies with three arms reporting on 10 different EOs (20 arms on *lavender*, 11 *damask rose*, 9 *citrus aurantium L*., two arms each on *lippia alba, mint*, and *lemon*, one arm each on *lippia citriodora, geranium, jasmine*, and *copaiba*, and 50 arms were control groups). The network formed by the direct comparisons between different interventions was shown in Figure 7A.

Comparing the DIC of the consistency and inconsistency models revealed that the consistency model should be used for the analysis (DIC_consistency_ = 183.12, DIC_inconsistency_ = 183.20).

**Figure 8:**
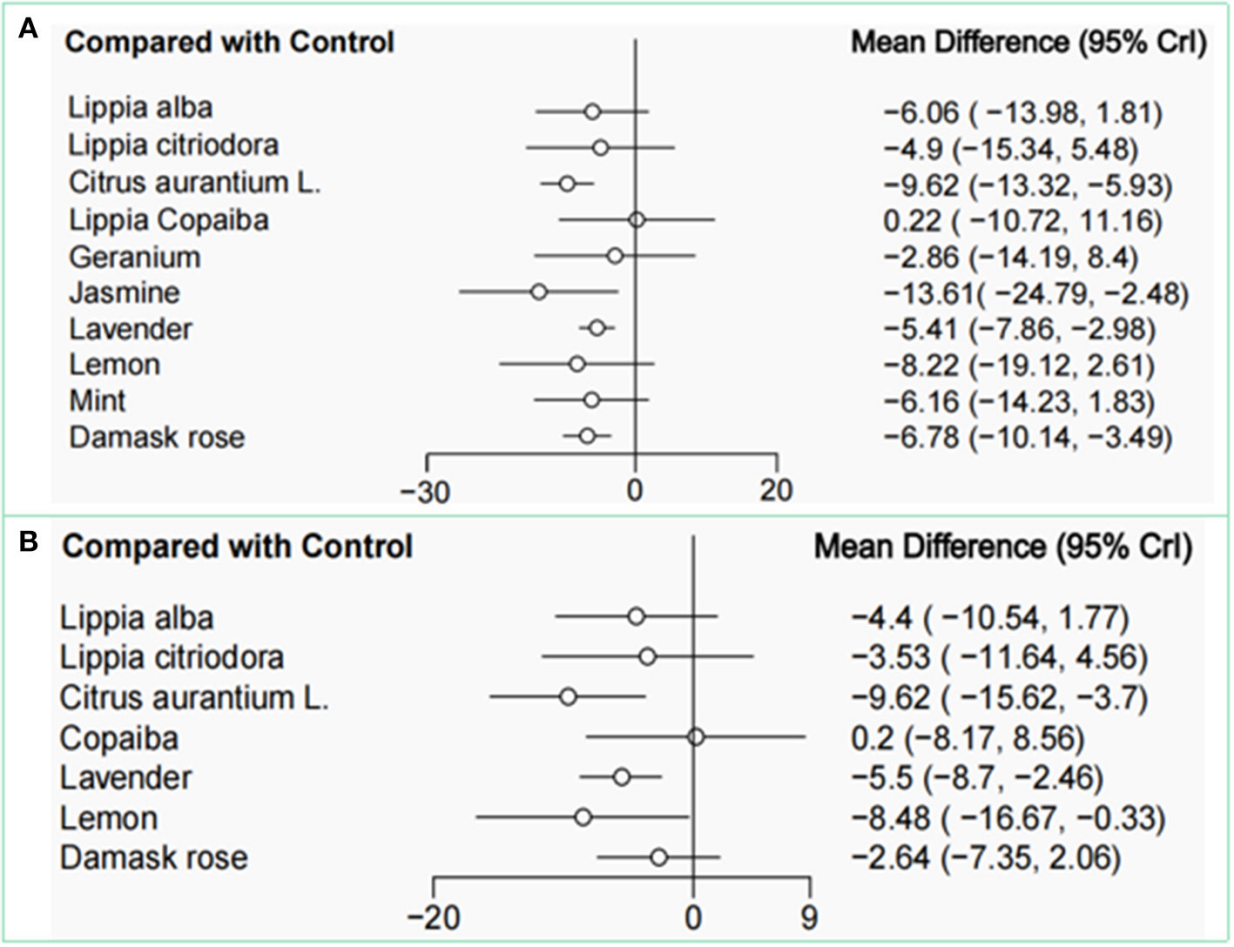
Network meta-analysis for comparisons with the control group of SAIS **(A)** and TAIS **(B)**. SAIS, State Anxiety Inventory scores; TAIS, Trait Anxiety Inventory scores.

The effect sizes for the differences between all EOs were presented in league table (Table 2). Figure 8A presented the findings of the indirect-comparisons meta-analysis as effect sizes and their 95% CrI for the different types of EOs interventions on SAIS compared with control group. Four (40%) among 10 interventions significantly decreased SAIS compared with the control group. *Jasmine* was the best ranked intervention with a WMD of−13.61 (95% CrI−24.79, −2.48) for state anxiety. Second only to *jasmine* is *citrus aurantium L*., which had an effect size of−9.62 (95% CrI−13.32, −5.93). Moderate effect sizes were observed for *damask rose* (WMD = −6.78, 95% CrI−10.14, −3.49) and *lavender* (WMD = −5.41, 95% CrI−7.86, −2.98), compared with the control group. The following interventions had credible intervals including a zero effect, including *mint* (WMD = −6.16, 95% CrI−14.23, 1.83), *lippia alba* (WMD = −6.06, 95% CrI−13.98, 1.81), *lemon* (WMD = −8.22, 95% CrI−19.12, 2.61), *lippia citriodora* (WMD = −4.9, 95% CrI−15.34, 5.48), and *geranium* (WMD = −2.86, 95% CrI−14.19, 8.4).

**Table 2:** Network meta-analysis diagram of SAIS and TAIS.

**Treatment**	**Trait anxiety inventory scores**											
		Jasmine	–	–	–	–	–	–	–	–	–	–
		−3.98 (−15.71, 7.71)	*Citrus aurantium* L.	6.98 (−0.48, 14.54)	–	5.24 (−3.36, 13.89)	4.11 (−2.19, 10.41)	5.24 (−3.36, 13.89)	1.13 (−8.93, 11.35)	–	9.82 (−0.38, 20.18)	9.62 (3.7, 15.62)
		−6.82 (−18.45, 4.79)	−2.84 (−7.75, 2.13)	Damask rose	–	−1.76 (−9.5, 6.07)	−2.87 (−8.19, 2.36)	−1.76 (−9.5, 6.07)	−5.84 (−15.26, 3.6)	–	2.84 (−6.72, 12.46)	2.64 (−2.06, 7.35)
**State anxiety inventory scores**
		−7.45 (−21.19, 6.22)	−3.46 (−12.28, 5.39)	−0.63 (−9.33, 8.07)	Mint	–	–	–	–	–	–	–
		−7.51 (−21.16, 6.13)	−3.57 (−12.22, 5.14)	−0.71 (−9.28, 7.84)	−0.09 (−11.31, 11.15)	Lippia alba	−1.11 (−8.12, 5.73)	0.85 (−7.32, 9)	−4.08 (−14.37, 6.2)	–	4.61 (−5.84, 15.05)	4.4 (−1.77, 10.54)
		−8.18 (−19.61, 3.18)	−4.22 (−8.43, 0.05)	−1.38 (−5.27, 2.48)	−0.76 (−9.12, 7.64)	−0.64 (−8.94, 7.59)	Lavender	1.11 (−5.73, 8.12)	−2.97 (−11.68, 5.83)	–	5.71 (−3.16, 14.7)	5.5 (2.46, 8.7)
		−8.69 (−24.01, 6.56)	−5.61 (−16.54, 5.23)	−1.88 (−12.82, 9.02)	−1.25 (−14.41, 11.83)	−1.15 (−11.55, 9.2)	−0.51 (−11.18, 10.23)	Lippia citriodora	−4.95 (−16.52, 6.55)	–	3.73 (−7.89, 15.41)	3.53 (−4.56, 11.64)
		−5.37 (−20.88, 10.29)	−1.38 (−12.85, 10.07)	1.45 (−9.94, 12.81)	2.07 (−11.45, 15.62)	2.17 (−11.23, 15.56)	2.83 (−8.34, 13.95)	3.31 (−11.72, 18.46)	Lemon	–	8.69 (−2.99, 20.43)	8.48 (0.33, 16.67)
		−10.76 (−26.63, 5.22)	−6.75 (−18.62, 5.13)	−3.91 (−15.68, 7.85)	−3.31 (−17.14, 10.55)	−3.2 (−17.01, 10.55)	−2.55 (−14.09, 9.05)	−2.06 (−17.39, 13.3)	−5.37 (−21.06, 10.31)	Geranium	–	–
		−13.82 (−29.47, 1.75)	−9.84 (−21.4, 1.7)	−7.01 (−18.42, 4.37)	−6.38 (−19.93, 7.24)	−6.28 (−19.8, 7.24)	−5.63 (−16.84, 5.58)	−5.13 (−20.27, 9.97)	−8.46 (−23.9, 6.97)	−3.08 (−18.85, 12.56)	Copaiba	−0.2 (−8.56, 8.17)
		−13.61 (−24.79, −2.48)	−9.62 (−13.32, −5.93)	−6.78 (−10.14, −3.49)	−6.16 (−14.23, 1.83)	−6.06 (−13.98, 1.81)	−5.41 (−7.86, −2.98)	−4.9 (−15.34, 5.48)	−8.22 (−19.12, 2.61)	−2.86 (−14.19, 8.4)	0.22 (−10.72, 11.16)	Control

SAIS, State Anxiety Inventory scores; TAIS, Trait Anxiety Inventory scores. The orange represents the different types of essential oils. The green represents the effect size of essential oils that significantly decrease SAIS or TAIS.

**Figure 9:**
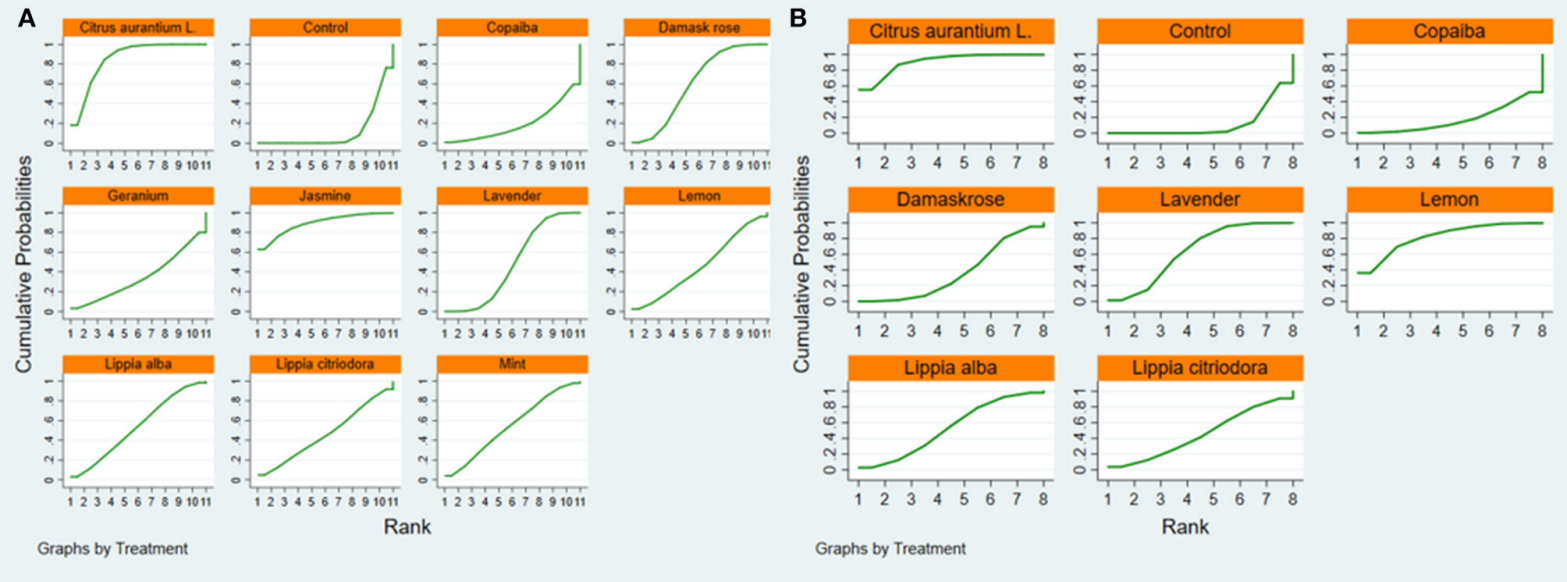
SUCRA figure of SAIS **(A)** and TAIS **(B)**. Note: SUCRA = surface under the cumulative ranking. SAIS, State Anxiety Inventory scores; TAIS, Trait Anxiety Inventory scores.

**Figure 10:**
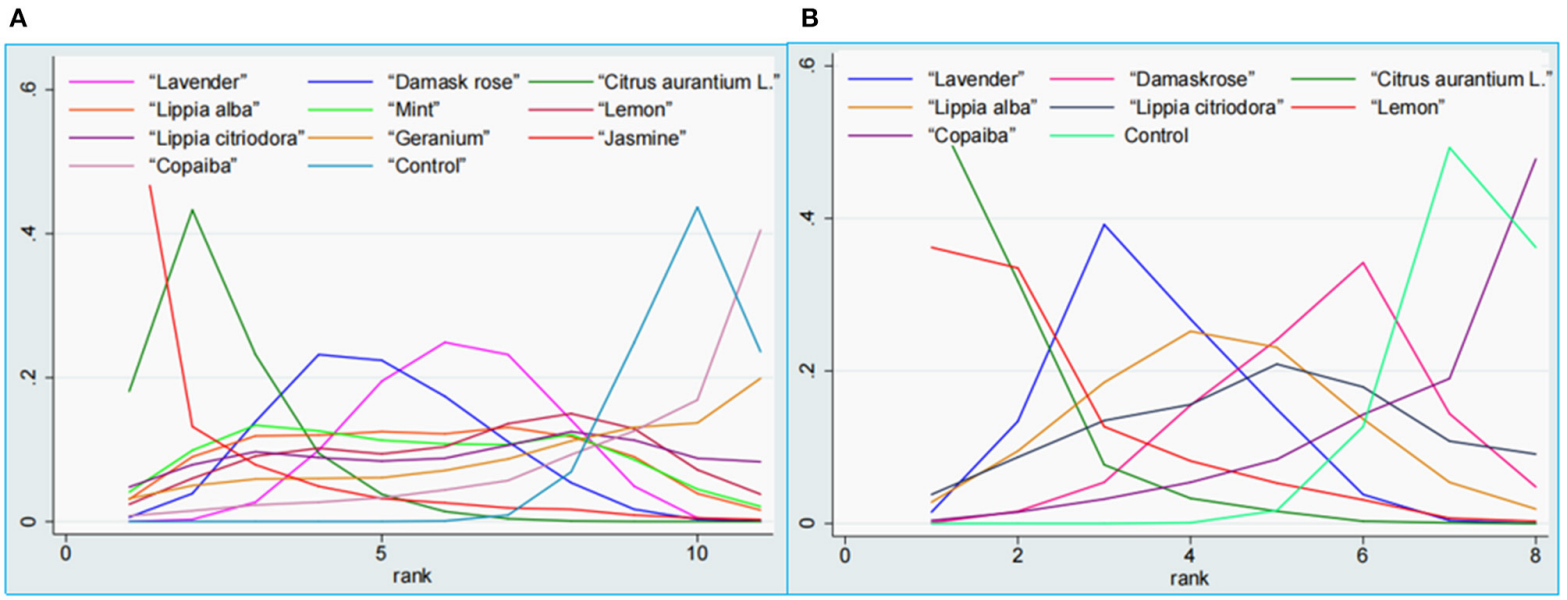
Ranking probability plot of SAIS **(A)** and TAIS **(B)**. SAIS, State Anxiety Inventory scores; TAIS, Trait Anxiety Inventory scores.

As shown in Table 3, the SUCRA indicated that *jasmine* was the best ranked intervention with a SUCRA of 88.1. The control intervention had the lowest SUCRA of 11.6 (Figures 9, 10).

**Table 3:** Ranking of values of SUCRA for SAIS and TAIS.

**Treatment**	**SUCRA**	**PrBest**	**MeanRank**
**SAIS**			
Control	11.6	0	9.8
Lavender	45.4	0	6.5
Damaskrose	57.9	0.5	5.2
CitrusaurantiumL	79.3	8.7	3.1
Lippiaalba	52	3.1	5.8
Mint	53.2	4.1	5.7
Lemon	64.5	15.6	4.6
Lippiacitriodora	45.3	4.8	6.5
Geranium	33.9	3.2	7.6
Jasmine	88.1	59.1	2.2
Copaiba	19	0.7	9.1
**TAIS**			
Control	11.5	0	7.2
Lavender	63.8	1.5	3.5
Damaskrose	36.3	0.1	5.5
CitrusaurantiumL	90.6	55.2	1.7
Lippia alba	53.1	2.8	4.3
Lippia citriodora	45.3	3.8	4.8
Lemon	81.9	36.2	2.3
Copaiba	17.4	0.4	6.8

SAIS, State Anxiety Inventory scores; TAIS, Trait Anxiety Inventory scores.

There are 3 loops were formed between the pairwise comparisons, and the loop inconsistency test indicated that there was no significant loop inconsistency (Table 4). The node-splitting model test showed no statistical local inconsistency between direct and indirect comparisons (*P* > 0.1). However, the overall heterogeneity remained high (93.2%), indicating that inconsistency did not explain the heterogeneity.

**Table 4:** Loop-specific approach.

**Outcomes**	**Loop**	**IF**	**seIF**	***z*-value**	***p*-value**	**CI_95**	**Loop_Heterog_tau^2^**
SAIS	Con-Lav-Dam	2.14	4.02	0.79	0.43	(0.00, 10.06)	20.88
	Con-Lav-Cit	1.33	4.15	0.32	0.75	(0.00, 9.46)	31.66
	Con-Alb-Citrio	0.16	2.81	0.06	0.96	(0.00, 5.67)	0.00
TAIS	Con-Lav-Dam	4.87	4.43	1.10	0.27	(0.00, 13.55)	10.06
	Con-Lav-Cit	4.85	5.03	0.96	0.34	(0.00, 14.70)	11.32
	Con-Alb-Citrio	0.15	2.97	0.05	0.96	(0.00, 5.96)	0.00

SAIS, State Anxiety Inventory scores; TAIS, Trait Anxiety Inventory scores; Con, Control; Lav, Lavender; Dam, Damask rose; Cit, Citrus aurantium L.; Alb, Lippia alba; Citrio, Lippia citriodora.

#### 3.5.2. Indirect-comparisons meta-analysis for EOs on trait anxiety scores

This network consisted of 14 RCTs, including 11 studies with two arms and 3 studies with three arms, involving 7 different EOs (7 *lavender*, 3 *damask rose*, two arms each on *citrus aurantium L*. and *lippia alba*, one arm each on *lippia citriodora, lemon*, and *copaiba*, and 17 arms were control groups). The most applied EO was *lavender*. The network formed by the direct comparisons between different interventions was shown in Figure 7B.

Comparing the DIC for the consistency and inconsistency models revealed that the consistency model was to be preferred (DIC_consistency_ = 60.77, DIC_inconsistency_ = 60.84).

The effect sizes for the differences between all EOs on TAIS were presented in league table (Table 2). Figure 8B presented the findings of the indirect-comparisons meta-analysis as effect sizes and their 95% CrI for the different types of EOs interventions on trait anxiety scores (TAS) compared with control group. Three (43%) among 7 interventions significantly decreased TAS compared with the control group. *Citrus aurantium L*. was the highest ranked intervention with a WMD of −9.62 (95% CrI−15.62, −3.7) for trait anxiety. Moderate-to-large effect sizes were observed for *lemon* (WMD: −8.48; 95% CrI−16.67, −0.33) and *lavender* (WMD: −5.5; 95% CrI−8.7, −2.46). The following 4 interventions had credible intervals including a zero effect, including *lippia alba* (WMD = −4.4, 95% CrI−10.54, 1.77), *lippia citriodora* (WMD = −3.53, 95% CrI−11.64, 4.56), *damask rose* (WMD = −2.64, 95% CrI−7.35, 2.06), and *copaiba* (WMD = 0.2, 95% CrI−8.17, 8.56).

As shown in Table 3, the SUCRA indicated that *citrus aurantium L*. was the highest ranked intervention with a SUCRA of 90.6. The control intervention had the lowest SUCRA of 11.5 (Figures 9, 10).

There are 3 loops were formed between the pairwise comparisons, and the loop inconsistency test indicated that there was no significant loop inconsistency (Table 4). The node-splitting model test confirmed statistical inconsistency between local direct and indirect comparisons (*P* < 0.1), suggesting that inconsistency may explain the overall high heterogeneity (I^2^ = 93.9%).

### 3.6. Subgroup and meta-regression analysis

Subgroup analyses stratified by type of EOs, country, causes of anxiety, and cumulative duration of intervention were performed (Supplementary Table S5 and Figure S5).

According to the results of subgroups by types of EOs, most EOs could significantly reduce SAIS, TAIS, SBP, DBP, HR or RR. However, *citrus aurantium L*. had no significant effect on RR [WMD = −1.402 (95% CI−3.066, 0.263), *P* = 0.099]. *Lemon* could not significantly reduce SAIS [WMD = −8.24 (95% CI−10.16, −6.32), *P* = 0.167] and DBP [WMD = 1.740 (95% CI−2.018, 5.498), *P* = 0.364]. *Mint* failed to reduce SBP [WMD = −0.660 (95% CI−8.673, 7.353), *P* = 0.872], DBP [WMD = 0.530 (95% CI−2.513, 3.573), *P* = 0.733] and HR [WMD = 0.290 (95% CI−0.969, 1.549), *P* = 0.652]. *Lavender* failed to reduce DBP [WMD = −1.627 (95% CI−4.655, 1.401), *P* = 0.292] and RR [WMD = 0.039 (95% CI−0.493, 0.570), *P* = 0.887], and *geranium* had no significant improvement on SAIS [WMD = −2.88 (95% CI−6.40, 0.64), *P* = 0.108], SBP [WMD = 0 (95% CI−7.151, 7.151), *P* = 1], DBP [WMD = −5 (95% CI−10.614, 0.6144), *P* = 0.081] and HR [WMD = 2.000 (95% CI−2.605, 6.605), *P* = 0.395]. *Copaiba* had the weakest efficacy and could not reduce SAIS [WMD = 0.20 (95% CI−2.09, 2.49), *P* = 0.864] and TAIS [WMD = 0.20 (95% CI−2.09, 2.49), *P* = 0.395].

With respect to the country, studies from Iran, Peru, and Turkey found that EOs could significantly reduce SAIS and TAIS. Studies in Brazil and Philippines have only reported that EOs reduced SAIS. However, Turkish studies concluded that EOs had no significant effect on SBP [WMD = −3.527 (95% CI−8.479, 1.425), *P* = 0.163] and HR [WMD = −3.498 (95% CI−9.246, 2.251), *P* = 0.233]. According to the studies of Singapore, EOs could not reduce SAIS [WMD = −3.7 (95% CI−8.465, 1.065), *P* = 0.128], SBP [WMD = −2.880 (95% CI−11.724, 5.964), *P* = 0.523] and HR [WMD = −2.3 (95% CI−7.664, 3.064), *P* = 0.401], and the Chinese study believed that EOs could not reduce SAIS [WMD = −0.033 (95% CI−1.512, 1.445), *P* = 0.965], TAIS [WMD = 0.2 (95% CI−2.090, 2.490), *P* = 0.864], SBP [WMD = −4.050 (95% CI−19.770, 11.670), *P* = 0.614] and HR [WMD = −0.150 (95% CI−9.996, 9.696), *P* = 0.976]. Interestingly, only the Brazilian study concluded that EOs significantly reduced DBP [WMD = −6.4 (95% CI−7.886, −4.914), *P* < 0.001] and RR [WMD = −2.3 (95% CI−2.605, −1.995), *P* < 0.001], while other countries found that EOs had no obvious improvement in them.

In causes of anxiety respect, our results showed that EOs could not reduce SAIS in patients with burn-related anxiety [WMD = −15.13 (95% CI−20.354, −9.906), *P* = 0.481] and TAIS in menopause-related anxiety [WMD = −0.650 (95% CI−2.849, 1.549), *P* = 0.562]. EOs were poorly efficacious in delivery-related anxiety, failing to reduce TAIS, SBP, DBP, HR, and RR. Similarly, EOs had no significant effect on TAIS [WMD = −3.250 (95% CI−12.315, 5.815), *P* = 0.482], DBP [WMD = −2.745 (95% CI−5.952, 0.462), *P* = 0.093], and RR [WMD = −0.362 (95% CI−2.698, 1.974), *P* = 0.761] in patients with invasive examination-related anxiety. EOs could not improve the physiological parameters of operation-related anxiety, including SBP [WMD = −4.256 (95% CI−9.159, 0.648), *P* = 0.089], DBP [WMD = −0.072 (95% CI−2.977, 2.832), *P* = 0.961], HR [WMD = −1.887 (95% CI−5.508, 1.735), *P* = 0.307], and RR [WMD = −0.434 (95% CI−1.249, 0.381), *P* = 0.296]. Additionally, EOs did not have apparent effects on SBP [WMD = −16.859 (95% CI−48.747, 15.029), *P* = 0.3], DBP [WMD = 1.009 (95% CI−1.356, 3.374), *P* = 0.403], and HR [WMD = −6.528 (95% CI−20.243, 7.187), *P* = 0.351] in cardiovascular disease-induced anxiety.

Regarding the cumulative duration of intervention, we found that the general efficacy of EOs for anxiety was optimal when the cumulative duration of intervention was 10~30 min, which could not only effectively reduce SAIS [WMD = −5.815 (95% CI−7.779, −3.851), *P* < 0.001] and TAIS [WMD = −4.935 (95% CI−7.882, −1.989), *P* < 0.001], but also stabilize vital signs, including lowering SBP [WMD = −5.372 (95% CI−8.484, −2.259), *P* = 0.001], DBP [WMD = −3.235 (95% CI−6.002, −0.467), *P* = 0.022], and HR [WMD = −3.184 (95% CI−5.050, −1.318), *P* = 0.001]. In fact, EOs reduced SAIS regardless of the duration of the intervention, as evidenced by further meta-regression analysis (*P* > 0.05). However, EOs had no significant effect on SBP, DBP, and HR when the cumulative duration of intervention was < 10 min or maintained at 30~100 min, and had no improvement on TAIS when it was >500 min [WMD = −4.625 (95% CI−12.307, 3.057), *P* = 0.238]. Meta-regression analysis confirmed that the effect size of EOs in lowering DBP was significantly negatively correlated with the intervention time when it was within 30 min (regression = −2.70, *P* = 0.027). Additionally, the effect size of EOs in lowering SBP and HR also showed a negative trend with the intervention time when it was controlled within 30 min, but it was not statistically significant (*P* > 0.05) (Table 5).

**Table 5:** Meta-regression of weighted mean difference according to cumulative duration of intervention.

**Effect size**	**Coef**.	**Std. Err**.	**t**	**P>|t|**	**95% CI**	**tau^2^**	**Adj R-squared (%)**
SAIS	−0.003	0.002	−1.42	0.163	−0.007~0.001	25.34	1.68
TAIS	−0.001	0.002	−0.58	0.571	−0.005~0.003	15.01	−4.82
SBP	−0.178	0.162	−1.10	0.304	−0.550~0.195	7.131	13.88
DBP	−0.296	0.110	−2.70	0.027	−0.549~-0.043	3.789	58.81
HR	−0.137	0.131	−1.05	0.326	−0.439~0.165	5.275	−15.56

SAIS, State Anxiety Inventory scores; TAIS, Trait Anxiety Inventory scores; SBP, systolic blood pressure; DBP, diastolic blood pressure; HR, heart rate.

### 3.7. Sensitivity analysis

In the sensitivity analyses to test the effect of smaller sample sizes on the overall weighted mean in the “State Anxiety Inventory” studies, the effect sizes of most comparisons were decreased. The overall effect size for EO interventions on SAIS was reduced from −6.63 to −0.84 when excluding smaller studies, although none of the confidence intervals included the zero effect. Additionally, the exclusion of small studies did not generate a reduction in heterogeneity (Supplementary Figure S6A).

Interestingly, in the sensitivity analyses to test the effect of smaller sample sizes on the overall weighted mean, studies using the Trait Anxiety Inventory as an evaluator showed the same effect size trends as the State Anxiety Inventory. The overall effect size for EO interventions on TAIS was reduced from −4.97 to −1.51 when excluding smaller studies, and none of their credible intervals included the zero effect. The exclusion of small studies did not decrease the heterogeneity (Supplementary Figure S6B).

### 3.8. Publication bias

Supplementary Figure S7 presented the funnel plot of the publication bias. The distribution of most dots in the figure was relatively symmetrical and uniform, indicating little evidence of publication bias. However, there are some dots distributed outside of the 95% CI, suggesting the effect of small sample size may exist.

### 3.9. Certainty of evidence

CINeMA application was used to conduct GRADE judgments. The therapeutic effect of EO on anxiety was measured by SAI inventory, twenty-six of comparisons were judged to be low rating, with twenty-nine very low comparisons while measured by TAI inventory, eighteen of comparisons were judged as low rating, with ten very low comparisons (Supplementary Tables S3.1, S3.2).

## 4. Discussion

### 4.1. Main study findings

To the best of our knowledge, this was the first study about different EOs for treating anxiety using Bayesian network meta-analysis. Anxiety was assessed with the Spielberger State-Trait Anxiety Inventory (STAI), which is currently the most extensively used inventory for evaluating anxiety levels and consists of two parts, SAI and TAI. The changes of SAIS and TAIS were used as the primary outcomes to evaluate the therapeutic effects of EOs on anxiety in this study. Pairwise meta-analyses indicated that EOs could effectively reduce SAIS and TAIS. Further network meta-analyses showed that *jasmine* was the most effective EO in reducing SAIS, followed by *citrus aurantium L*., which ranked first in reducing TAIS.

Vital signs are considered to be important physiological indicators of anxiety indirectly (13). Emotional signals of anxiety are sent from the amygdala and hippocampus of the limbic system to the hypothalamus, activating the hypothalamic-pituitary-adrenocortical (HPA) axis (61). Moreover, anxiety could cause increased sympathetic excitability, which leads to increased blood pressure, HR and RR. Herein, vital signs may be helpful in objective assessment of anxiety. Our results found that EOs dramatically reduced SBP and HR, and had a tendency to lower DBP and RR, but were not statistical significance.

### 4.2. Possible explanation for study findings

Our findings were consistent with previous conventional meta-analyses focusing on EOs for anxiety. Indicating that EOs significantly alleviate anxiety (62, 63). Additionally, our results further confirmed the results of a previous meta-analysis pooling animal experiments from a clinical perspective (64).

Pairwise meta-analyses suggested that EOs had a stronger total therapeutic effect on state anxiety than trait anxiety. This was consistent with the results of an earlier meta-analysis (65). Moreover, we further found that EOs were effective in reducing state anxiety regardless of the cumulative duration of the intervention. However, EOs could not alleviate trait anxiety when the cumulative intervention time exceeded 500 min, with the largest effect size within 10 min of the intervention time. Thus, the efficacy of EOs on trait anxiety was more inclined to immediate intervention. The reason for these results may be related to the mechanisms of state anxiety and trait anxiety. Previous study has shown that state anxiety and trait anxiety are mapped differently in the brain (66). State anxiety is a transient intense emotional state associated with a temporary increase in sympathetic nervous system activity without a specific pathological condition, and it can disappear with the removal of stress or danger (66). Trait anxiety is a personality tendency that remains stable over time (67). It may be associated with different psychopathological conditions and continuous high arousal. It does not disappear easily when the stress is relieved, and people with high trait anxiety are easy to develop anxiety disorders (68). Therefore, EOs have a more obvious therapeutic effect on state anxiety. Trait anxiety symptoms could restore to their stable personality characteristics soon, although a short period of EOs stimulation can effectively alleviate them.

Network meta-analyses suggested that that *jasmine* was the strongest type of EO for reducing SAIS. Contrary to previous studies that suggested that *jasmine* could only improve nervousness and not reduce symptoms of anxiety and stress (58), our study found that *jasmine* significantly improved state anxiety symptoms and thus reduced SAIS. Pharmacological studies suggested that the mechanisms by which *jasmine* reduced SAIS may be related to the increase of β wave in the frontal cortex center and left occipital cortex caused by olfactory stimulation (69). However, only one study explored the efficacy of *jasmine* on anxiety, so the credibility of this result is low and more researches are indispensable to confirm this result.

Considering that there was only one RCT reporting *jasmine* for anxiety, the results may be highly biased and less credible. *Citrus aurantium L*. (85.6%) and *jasmine* (89.3%) had similar efficacy in reducing SAIS according to SUCRA values, and *citrus aurantium L*. ranked first in reducing TAIS. Furthermore, *citrus aurantium L*. could dramatically reduce the objective indicators reflecting anxiety, including SBP, DBP, and HR. The anxiolytic activity of *citrus aurantium L*. was mediated by the serotonergic system (5-HT1A receptor) (7, 70). Interestingly, *citrus aurantium L*. significantly ameliorated anxiety and did not interfere with physiological levels of melatonin and corticosterone (71). Thus, it could be reasonably inferred that *citrus aurantium L*. EO has the greatest benefits in treating anxiety.

Previous studies on the treatment of anxiety with lemon EO are still somewhat controversial. An RCT investigating the effect of lemon EO on anxiety during the active phase in primiparas showed that lemon EO had no effect on anxiety (72). However, another multicenter, assessor-blinded trial demonstrated the efficacy of aromatherapy with lemon EO inhalation in relieving anxiety (43). To the best of our knowledge, this study was the first meta-analysis of lemon EO for anxiety. Our paired meta-analysis confirmed that lemon EO significantly ameliorated trait anxiety, but did not alleviate state anxiety. Further, our network meta-analysis found that *lemon* ranked second among all EOs in the aspect of reducing TAIS. The main component of *lemon* is s-limonene. The anti-trait anxiety effect of *lemon* is closely related to the 5-serotonergic pathway, particularly through the 5-HT (1A) receptor. In addition, *lemon* significantly accelerated the metabolic turnover of dopamine (DA) in the hippocampus and 5-HT in the prefrontal cortex and striatum (69).

Current network meta-analyses also found that *damask rose* was the third most anti-anxiety EO after *jasmine* and *citrus aurantium L*. in reducing SAIS. However, *damask rose* could not reduce TAIS. This finding was in line with a previous meta-analysis that pooled the studies about *damask rose* mill on anxiety, indicating that *damask rose* dramatically decreased state anxiety but had no obvious effect on trait anxiety (73). *Damask rose* mainly contains isoflavones. On one hand, isoflavones can directly bind to GABA receptors to reduce anxiety (74). On the other hand, isoflavones inhibit inducible nitric oxide synthase (iNOS) and then reduce the production of nitric oxide, which regulates the concentration of neurotransmitters such as serotonin, dopamine, norepinephrine and glutamate, and inhibits the activation of soluble guanylate cyclase, which in turn reduces the production of cyclic guanosine monophosphate (cGMP), thereby reducing anxiety (75).

The number of studies using *lavender* as an intervention were the largest for both state anxiety and trait anxiety. Our results indicated that the efficacy of *lavender* in reducing SAIS and TAIS was comparable. This was consecutive with the results of previous meta-analyses (12, 13). Researches in pharmacology have found that the anxiolytic effects of *lavender* are related to the interaction of its monoterpene components such as linalool and linalyl acetate with NMDA receptors. Additionally, the anxiolytic effect could be partially attributed to its inhibition of serotonin transporter (SERT) and protection of SH-SY5Y cells from hydrogen peroxide-induced neurotoxicity (76).

Results of an RCT using *mint* intervention in emergency cardiac patients found that *mint* reduced anxiety and its induced increase in respiratory rate. In keeping with this, our study indicated that *mint* could significantly reduce SAIS. *Mint* affects the hypothalamus by stimulating the olfactory pathway, which reduces the secretion of adrenocorticotropin-releasing hormone, adrenocorticotropin and cortisol, ultimately reducing anxiety (77, 78).

Finally, the results of our study showed that neither *geranium* nor *copaiba* could reduce anxiety, and *copaiba* ranked the worst. However, the RCTs on *geranium* and *copaiba* for anxiety each only one was included, which tends to cause significant heterogeneity, so these results should be interpreted with caution and more studies are needed to confirm these conclusions in the future.

No adverse events were reported in any of the trials, and their safety remains uncertain. EOs gas molecules can be inhaled and then transported to the central nervous system after entering the bloodstream through the lung, or they can cross the neuronal network of the olfactory system and directly reach and act on the corresponding brain areas, causing activation in different brain regions to induce anxiolytic effects (8). Several studies concluded that EOs appeared to be well tolerated, as inhalation of different doses of EOs did not cause changes or show signs of toxicity (48).

In summary, the most effective was *jasmine* intervention with a cumulative duration of 30 to 100 min for state anxiety, while *citrus aurantium L*. intervention with a cumulative duration of 10 min or less were most efficacious for trait anxiety. Nevertheless, combining self-reported SAIS and TAIS with objective indicators, a cumulative intervention of 10 to 30 min with *citrus aurantium L*. was optimal, because it reduced not only SAIS and TAIS, but also SBP, DBP, and HR. However, it is worth noting that due to the lack of reports on adverse effects, the studies with EOs included in this study could not be fully used to rank the safety of EOs. This limitation requires special attention in future studies.

### 4.3. Strength and limitations

The current study has several strengths. We developed rigorous eligibility criteria, conducted a comprehensive search, assessed the risk of bias, addressed key outcomes, performed valuable sensitivity and subgroup analyses, and rated the certainty of the evidence using CINeMA by GRADE criteria. For the first time, a network meta-analysis was used to compare the differences in efficacy of various types of commonly used EOs, providing clinicians with a rational choice of essential oils for the treatment of anxiety.

However, the study also had a number of limitations. To comprehensively summarize the efficacy of EOs, we included a wide range of interventions that varied in terms of number of interventions, duration, dose, co-morbidities, or causes of anxiety, so that there may be large heterogeneity across trials. Moreover, most of the trials had a high risk of bias and low or very low quality of evidence. Therefore, the credibility of the results was low and we need to be cautious in interpreting the results. In addition, the intrinsic characteristics of EOs, such as the species, place of origin, and the intervention procedures of EOs, including extraction method, exposure concentration, method of exposure, duration of exposure, and route of administration, could affect the amount of active ingredients of EOs in the body and thus the efficacy of EOs. Thus, only by controlling for these variables can the efficacy of EOs for anxiety in different clinical trials be more accurately determined. What's more, more high-quality RCTs that rigorously document the characteristics and intervention procedures of EO, may be needed in the future to improve the reliability of meta-analysis conclusions.

## 5. Conclusion

This study confirmed that EOs may be effective in treating anxiety, and *citrus aurantium L*. appeared to be the most recommended type, as it showed a large effect size in reducing both SAIS and TAIS. However, due to the obvious heterogeneity among the included studies, our results should be interpreted with caution and more high-quality RCTs are expected to confirm this result in the future.

## Data availability statement

The original contributions presented in the study are included in the article/Supplementary material, further inquiries can be directed to the corresponding authors.

## Author contributions

LT and F-fL: conceptualization, visualization, investigation, and writing-original draft. L-zL and C-gF: conceptualization, visualization, and writing – review and editing. X-cM: investigation and writing – review and editing. Y-xP: formal analysis and writing – review and editing. J-mL and HQ: funding acquisition and writing – review and editing. All authors contributed to the article and approved the submitted version.
