# Supplementary material for: Essential oils for treating anxiety: a systematic review of randomized controlled trials and network meta-analysis
Source: Front Public Health. 2023 Jun 1;11:1144404. doi: 10.3389/fpubh.2023.1144404 (PMC10267315; doi:10.3389/fpubh.2023.1144404)
Supplement: Supplementary file 1 [file Table_1.doc]

**Essential Oils for Treating Anxiety: A Systematic Review of Randomized Controlled Trials and Network Meta-analysis**

**Contents**

**Table S. PRISMA 2020 for Network Meta-Analyses Checklist.**

**Appendix 1 Search Strategy**

**Appendix 2 Risk of Bias**

**Appendix 3 Grading the evidence of the NMA using CINeMA**

Table S3 Efficacy by SAI measure for anxiety.

Figure S3.1. Efficacy by SAI measure for anxiety.

Figure S3.2. Efficacy by SAI measure for anxiety.

**Appendix 4 Network plot-All trials**

Figure S4 Inconsistency test for closed loops.

Table S4. The ranking probabilities for each type of essential oil.

**Appendix 5 Results of subgroup analysis for various outcomes**

Table S5. Meta-analyses of essential oils on anxiety and physiological parameters.

Figure S5. The Forest plots for subgroup analyses.

**Appendix 6 Sensitivity analysis**

**Appendix 7 Publication Bias**

| **Abbreviation** | **Full name** |
| --- | --- |
| EO | Essential oil |
| SAI | State Anxiety Inventory |
| TAI | Trait Anxiety Inventory |
| STAI | Spielberger State-Trait Anxiety Inventory |
| SAIS  TAIS  STAIS | State Anxiety Inventory score  Trait Anxiety Inventory score  Spielberger State-Trait Anxiety Inventory score |
| SBP | Systolic blood pressure |
| DBP | Diastolic blood pressure |
| HR | Heart rate |
| RR | Respiratory rate |
| BDZ | Benzodiazepines |
| SSRIs | Selective serotonin reuptake inhibitors |
| CAM | Complementary and Alternative Medicine |
| RCT | Randomized Controlled Trials |
| NMA | Network meta-analysis |
| PMA | Pairwise meta-analysis |
| MD | Mean difference |
| WMD | Weighted mean difference; |
| OR | Odds ratio |
| CI | Confidence interval |
| CrI | Credible interval |
| RoB | Risk of bias |
| DIC | Deviance Information Criterion |

***Appendix 1 Search Strategy***

**Table S1 Search strategy for five electronic databases**

| Database | Search strategy | No. |
| --- | --- | --- |
| PubMed | #1: "anxiety"[MeSH]  #2: "angst*"[tiab] OR "hypervigilance*"[tiab] OR "nervousness*"[tiab] OR "anxiousness*"[tiab] OR "affect*"[tiab] OR "mood*"[tiab] OR "emotion*"[tiab] OR "phobia*"[tiab] OR "patient health questionnaire*"[tiab] OR "PHQ*"[tiab] OR "GAD*"[tiab] OR "panic*"[tiab] OR "attacks*"[tiab] OR "generalized anxiety disorder scale*"[tiab] OR "PRIME-MD *"[tiab]  #3: #1 OR #2  #4: "oils, volatile"[MeSH]  #5: "volatile oils"[tiab] OR "essential oil"[tiab] OR "volatile oil"[tiab] OR "aromatherapy"[tiab] OR "aromatherapies"[tiab] OR "therapies, aroma"[tiab] OR "therapy, aroma"[tiab] OR "odorants"[tiab] OR "odorant"[tiab] OR "aroma"[tiab] OR "scent"[tiab] OR "fragrance"[tiab] OR "odor"[tiab]  #6: #4 OR #5  #7: "randomised controlled trial"[tiab] OR "RCT"[tiab] OR "RCTs"[tiab] OR "random"[tiab] OR "randomization"[tiab] OR "randomisation"[tiab] OR "randomly"[tiab] OR "randomised"[tiab] OR "randomized"[tiab] OR "clinical trial"[tiab] OR "randomized trial"[tiab] OR "controlled clinical trial"[tiab] OR "clinical observation"[tiab] OR "Placebo"[tiab]  #8: #3 AND #6 AND #7 | 416 |
| Web of Science -All Databases | #1: TS=("anxiety" OR "angst*" OR "hypervigilance*" OR "nervousness*" OR "anxiousness*" OR "affect*" OR "mood*" OR "emotion*" OR "phobia*" OR "patient health questionnaire*" OR "PHQ*" OR "GAD*" OR "panic*" OR "attacks*" OR "generalized anxiety disorder scale*" OR "PRIME-MD *")  #2: TS=("volatile oils" OR "essential oil" OR "volatile oil" OR "aromatherapy" OR "aromatherapies" OR "therapies, aroma" OR "therapy, aroma" OR "odorants" OR "odorant" OR "aroma" OR "scent" OR "fragrance" OR "odor")  #3: TS=("randomised controlled trial" OR "RCT" OR "RCTs" OR "random” OR “randomization" OR "randomisation" OR "randomly" OR "randomised" OR "randomized" OR "clinical trial" OR "randomized trial" OR "controlled clinical trial" OR "clinical observation" OR "Placebo")  #4: #1 AND #2 AND #3 | 1860 |
| Embase | #1: 'anxiety'/exp  #2: 'angst':ab,ti OR 'hypervigilance':ab,ti OR 'nervousness':ab,ti OR 'anxiousness':ab,ti OR 'affect':ab,ti OR 'mood':ab,ti OR 'emotion':ab,ti OR 'phobia':ab,ti OR 'patient health questionnaire':ab,ti OR 'PHQ':ab,ti OR 'GAD':ab,ti OR 'panic':ab,ti OR 'attacks':ab,ti OR 'generalized anxiety disorder scale':ab,ti OR 'PRIME-MD':ab,ti  #3: #1 OR #2  #4: 'oils, volatile'/exp  #5: 'volatile oils':ab,ti OR 'essential oil':ab,ti OR 'volatile oil':ab,ti OR 'aromatherapy':ab,ti OR 'aromatherapies':ab,ti OR 'therapies, aroma':ab,ti OR 'therapy, aroma':ab,ti OR 'odorants':ab,ti OR 'odorant':ab,ti OR 'aroma':ab,ti OR 'scent':ab,ti OR 'fragrance':ab,ti OR 'odor':ab,ti  #6: #4 OR #5  #7: 'randomised controlled trial'/exp  #8: 'RCT':ab,ti OR 'RCTs':ab,ti OR 'random':ab,ti OR 'randomization':ab,ti OR 'randomisation':ab,ti OR 'randomly':ab,ti OR 'randomised':ab,ti OR 'randomized':ab,ti OR 'clinical trial':ab,ti OR 'randomized trial':ab,ti OR 'controlled clinical trial':ab,ti OR 'clinical observation':ab,ti OR 'Placebo':ab,ti  #9: #7 OR #8  #10: #3 AND #6 AND #9 | 661 |
| Cochrane | #1:(anxiety):ab,ti,kw OR (angst):ab,ti,kw OR (hypervigilance):ab,ti,kw OR (nervousness):ab,ti,kw OR (anxiousness):ab,ti,kw OR (affect):ab,ti,kw OR (mood):ab,ti,kw OR (emotion):ab,ti,kw OR (phobia):ab,ti,kw OR (patient health questionnaire):ab,ti,kw OR (PHQ):ab,ti,kw OR (GAD):ab,ti,kw OR (panic):ab,ti,kw OR (attacks):ab,ti,kw OR (generalized anxiety disorder scale):ab,ti,kw OR (PRIME-MD):ab,ti,kw  #2:(volatile oils):ab,ti,kw OR (essential oil):ab,ti,kw OR (volatile oil):ab,ti,kw OR (aromatherapy):ab,ti,kw OR (aromatherapies):ab,ti,kw OR (therapies, aroma):ab,ti,kw OR (therapy, aroma):ab,ti,kw OR (odorants):ab,ti,kw OR (odorant):ab,ti,kw OR (aroma):ab,ti,kw OR (scent):ab,ti,kw OR (fragrance):ab,ti,kw OR (odor):ab,ti,kw  #3:(randomised controlled trial):ab,ti,kw OR (RCT):ab,ti,kw OR (RCTs):ab,ti,kw OR (random):ab,ti,kw OR (randomization):ab,ti,kw OR (randomisation):ab,ti,kw OR (randomly):ab,ti,kw OR (randomised):ab,ti,kw OR (randomized):ab,ti,kw OR (clinical trial):ab,ti,kw OR (randomized trial):ab,ti,kw OR (controlled clinical trial):ab,ti,kw OR (clinical observation):ab,ti,kw OR (Placebo):ab,ti,kw  #4: #1 AND #2 AND #3 | 794 |
| Scopus | #1: "anxiety" OR "angst*" OR "hypervigilance*" OR "nervousness*" OR "anxiousness*" OR "affect*" OR "mood*" OR "emotion*" OR "phobia*" OR "patient health questionnaire*" OR "PHQ*" OR "GAD*" OR "panic*" OR "attacks*" OR "generalized anxiety disorder scale*" OR "PRIME-MD *"  #2: "volatile oils" OR "essential oil" OR "volatile oil" OR "aromatherapy" OR "aromatherapies" OR "therapies, aroma" OR "therapy, aroma" OR "odorants" OR "odorant" OR "aroma" OR "scent" OR "fragrance" OR "odor"  #3: "randomised controlled trial" OR "RCT" OR "RCTs" OR "random” OR “randomization" OR "randomisation" OR "randomly" OR "randomised" OR "randomized" OR "clinical trial" OR "randomized trial" OR "controlled clinical trial" OR "clinical observation" OR "Placebo"  #4: #1 AND #2 AND #3 | 1806 |

***Appendix 2 Risk of Bias***

**Table S2 Risk of bias summary.**

| **Study** | **Bias arising from the randomization process** | **Bias due to deviations from intended interventions** | **Bias due to missing outcome data** | **Measurement of the outcome** | **Bias in selection of the reported result** | **Overall risk of bias** | **Weight** |
| --- | --- | --- | --- | --- | --- | --- | --- |
| Abbasijahromi A. 2019 | some concern | low risk | low risk | some concern | low risk | low risk | 90 |
| Alvarado-García P.A.A. 2021 | low risk | high risk | low risk | some concern | low risk | high risk | 80 |
| Amzajerdi A. 2019 | low risk | some concern | low risk | low risk | low risk | low risk | 66 |
| Babatabar Darzi H. 2020 | low risk | some concern | high risk | some concern | some concern | high risk | 120 |
| Bahadori H. 2022 | low risk | some concern | low risk | some concern | low risk | low risk | 60 |
| Bakhsha F. 2014 | some concern | some concern | high risk | some concern | some concern | high risk | 100 |
| Beyliklioglu A. 2018 | some concern | high risk | low risk | some concern | low risk | high risk | 80 |
| Eslami J. 2021 | some concern | some concern | low risk | some concern | low risk | some concern | 60 |
| Eslami J. 2018 | some concern | some concern | low risk | some concern | low risk | some concern | 90 |
| Farzaneh M. 2022 | some concern | low risk | low risk | some concern | low risk | low risk | 38 |
| Fayazi S. 2011 | some concern | some concern | low risk | some concern | low risk | some concern | 72 |
| Ganji M.R. 2022 | low risk | some concern | low risk | some concern | low risk | low risk | 88 |
| Haddadi M. 2021 | some concern | some concern | low risk | some concern | low risk | some concern | 80 |
| Hamdamian S. 2017 | some concern | high risk | low risk | some concern | low risk | high risk | 110 |
| Hekmatpou D. 2017 | some concern | some concern | low risk | some concern | low risk | some concern | 60 |
| Hu PH. 2010 | some concern | some concern | low risk | some concern | low risk | some concern | 27 |
| Jirdehi M.M. 2022 | low risk | high risk | low risk | some concern | low risk | high risk | 105 |
| Jodaki K. 2021 | low risk | some concern | low risk | some concern | low risk | low risk | 60 |
| Jokar.M. 2020 | low risk | high risk | low risk | some concern | low risk | high risk | 62 |
| Kasar K.S. 2021 | some concern | some concern | low risk | some concern | low risk | some concern | 44 |
| Mokhtari R. 2022 | some concern | high risk | low risk | low risk | low risk | high risk | 60 |
| Moradi K. 2021 | some concern | high risk | low risk | some concern | low risk | high risk | 80 |
| Moslemi F. 2019 | some concern | some concern | low risk | low risk | low risk | low risk | 140 |
| Ozkaraman A. 2018 | some concern | low risk | low risk | low risk | low risk | low risk | 50 |
| Pasyar N. 2020 | low risk | some concern | low risk | some concern | low risk | low risk | 60 |
| Pimenta F.C.F. 2016 | some concern | some concern | high risk | some concern | low risk | high risk | 28 |
| Rambod M. 2020 | low risk | high risk | low risk | some concern | low risk | high risk | 110 |
| Reyes MCGM. 2020 | high risk | some concern | low risk | some concern | low risk | high risk | 50 |
| Sahin S. 2021 | some concern | some concern | low risk | some concern | low risk | some concern | 74 |
| Saritas S.C. 2021 | some concern | high risk | low risk | some concern | low risk | high risk | 90 |
| Shirzad M. 2022 | low risk | low risk | some concern | some concern | low risk | low risk | 68 |
| Soleimani M. 2022 | some concern | some concern | some concern | some concern | low risk | some concern | 64 |
| Soto-Vásquez M.R. 2018 | low risk | low risk | low risk | some concern | low risk | low risk | 55 |
| Stanley P.F. 2019 | some concern | some concern | low risk | some concern | low risk | some concern | 75 |
| Tahmasbi H. 2012 | some concern | some concern | low risk | some concern | low risk | some concern | 91 |
| Tahmasebi H. 2019 | low risk | some concern | low risk | some concern | low risk | low risk | 66 |
| Wen X.K. 2022 | low risk | low risk | low risk | some concern | low risk | low risk | 100 |
| Fakari F.R. 2015 | low risk | high risk | low risk | some concern | low risk | high risk | 97 |
| Babaii A. 2015 | some concern | high risk | low risk | some concern | low risk | high risk | 60 |
| Inci S. 2022 | low risk | some concern | low risk | some concern | low risk | low risk | 96 |
| Karan N.B. 2019 | low risk | some concern | low risk | some concern | low risk | low risk | 126 |
| Yadegari M. 2022 | low risk | high risk | low risk | some concern | low risk | high risk | 84 |
| Zhang N. 2022 | low risk | high risk | some concern | some concern | low risk | high risk | 22 |
| Tugut N. 2017 | low risk | some concern | low risk | some concern | low risk | low risk | 156 |

***Appendix 3: Grading the evidence of the NMA using CINeMA***

**Table S3****.1 Efficacy by SAI measure for anxiety.**

| **Comparison** | **Number of studies** | **Within-study bias** | **Reporting bias** | **Indirectness** | **Imprecision** | **Heterogeneity** | **Incoherence** | **Confidence rating** |
| --- | --- | --- | --- | --- | --- | --- | --- | --- |
| Citrus aurantium L.:Control | 10 | Some concerns | Low risk | No concerns | No concerns | Major concerns | No concerns | Low |
| Citrus aurantium L.:Lavender | 2 | Some concerns | Low risk | No concerns | No concerns | Major concerns | No concerns | Low |
| Control:Copaiba | 1 | Major concerns | Low risk | No concerns | Major concerns | No concerns | No concerns | Very low |
| Control:Damask rose | 11 | Some concerns | Low risk | No concerns | No concerns | Major concerns | No concerns | Low |
| Control:Geranium | 1 | Major concerns | Low risk | No concerns | Major concerns | No concerns | No concerns | Very low |
| Control:Jasmine | 1 | Major concerns | Low risk | No concerns | No concerns | Major concerns | No concerns | Very low |
| Control:Lavender | 20 | Some concerns | Low risk | No concerns | No concerns | Major concerns | No concerns | Low |
| Control:Lemon | 1 | Some concerns | Low risk | No concerns | Major concerns | No concerns | No concerns | Low |
| Control:Lippia alba | 2 | Major concerns | Low risk | No concerns | Major concerns | No concerns | No concerns | Very low |
| Control:Lippia citriodora | 1 | Major concerns | Low risk | No concerns | Major concerns | No concerns | No concerns | Very low |
| Control:Mint | 2 | Some concerns | Low risk | No concerns | Major concerns | No concerns | No concerns | Low |
| Damask rose:Lavender | 3 | Some concerns | Low risk | No concerns | Major concerns | No concerns | No concerns | Low |
| Lippia alba:Lippia citriodora | 1 | Major concerns | Low risk | No concerns | Major concerns | No concerns | No concerns | Very low |
| Citrus aurantium L.:Copaiba | 0 | Some concerns | Low risk | No concerns | Major concerns | No concerns | No concerns | Low |
| Citrus aurantium L.:Damask rose | 0 | Some concerns | Low risk | No concerns | Major concerns | No concerns | No concerns | Low |
| Citrus aurantium L.:Geranium | 0 | Some concerns | Low risk | No concerns | Major concerns | No concerns | No concerns | Low |
| Citrus aurantium L.:Jasmine | 0 | Some concerns | Low risk | No concerns | Major concerns | No concerns | No concerns | Low |
| Citrus aurantium L.:Lemon | 0 | Some concerns | Low risk | No concerns | Major concerns | No concerns | No concerns | Low |
| Citrus aurantium L.:Lippia alba | 0 | Some concerns | Low risk | No concerns | Major concerns | No concerns | No concerns | Low |
| Citrus aurantium L.:Lippia citriodora | 0 | Some concerns | Low risk | No concerns | Major concerns | No concerns | No concerns | Low |
| Citrus aurantium L.:Mint | 0 | Some concerns | Low risk | No concerns | Major concerns | No concerns | No concerns | Low |
| Copaiba:Damask rose | 0 | Major concerns | Low risk | No concerns | Major concerns | No concerns | No concerns | Very low |
| Copaiba:Geranium | 0 | Major concerns | Low risk | No concerns | Major concerns | No concerns | No concerns | Very low |
| Copaiba:Jasmine | 0 | Major concerns | Low risk | No concerns | Major concerns | No concerns | No concerns | Very low |
| Copaiba:Lavender | 0 | Major concerns | Low risk | No concerns | Major concerns | No concerns | No concerns | Very low |
| Copaiba:Lemon | 0 | Major concerns | Low risk | No concerns | Major concerns | No concerns | No concerns | Very low |
| Copaiba:Lippia alba | 0 | Major concerns | Low risk | No concerns | Major concerns | No concerns | No concerns | Very low |
| Copaiba:Lippia citriodora | 0 | Major concerns | Low risk | No concerns | Major concerns | No concerns | No concerns | Very low |
| Copaiba:Mint | 0 | Major concerns | Low risk | No concerns | Major concerns | No concerns | No concerns | Very low |
| Damask rose:Geranium | 0 | Major concerns | Low risk | No concerns | Major concerns | No concerns | No concerns | Very low |
| Damask rose:Jasmine | 0 | Major concerns | Low risk | No concerns | Major concerns | No concerns | No concerns | Very low |
| Damask rose:Lemon | 0 | Some concerns | Low risk | No concerns | Major concerns | No concerns | No concerns | Low |
| Damask rose:Lippia alba | 0 | Some concerns | Low risk | No concerns | Major concerns | No concerns | No concerns | Low |
| Damask rose:Lippia citriodora | 0 | Major concerns | Low risk | No concerns | Major concerns | No concerns | No concerns | Very low |
| Damask rose:Mint | 0 | Some concerns | Low risk | No concerns | Major concerns | No concerns | No concerns | Low |
| Geranium:Jasmine | 0 | Major concerns | Low risk | No concerns | Major concerns | No concerns | No concerns | Very low |
| Geranium:Lavender | 0 | Major concerns | Low risk | No concerns | Major concerns | No concerns | No concerns | Very low |
| Geranium:Lemon | 0 | Major concerns | Low risk | No concerns | Major concerns | No concerns | No concerns | Very low |
| Geranium:Lippia alba | 0 | Major concerns | Low risk | No concerns | Major concerns | No concerns | No concerns | Very low |
| Geranium:Lippia citriodora | 0 | Major concerns | Low risk | No concerns | Major concerns | No concerns | No concerns | Very low |
| Geranium:Mint | 0 | Major concerns | Low risk | No concerns | Major concerns | No concerns | No concerns | Very low |
| Jasmine:Lavender | 0 | Major concerns | Low risk | No concerns | Major concerns | No concerns | No concerns | Very low |
| Jasmine:Lemon | 0 | Major concerns | Low risk | No concerns | Major concerns | No concerns | No concerns | Very low |
| Jasmine:Lippia alba | 0 | Major concerns | Low risk | No concerns | Major concerns | No concerns | No concerns | Very low |
| Jasmine:Lippia citriodora | 0 | Major concerns | Low risk | No concerns | Major concerns | No concerns | No concerns | Very low |
| Jasmine:Mint | 0 | Major concerns | Low risk | No concerns | Major concerns | No concerns | No concerns | Very low |
| Lavender:Lemon | 0 | Some concerns | Low risk | No concerns | Major concerns | No concerns | No concerns | Low |
| Lavender:Lippia alba | 0 | Some concerns | Low risk | No concerns | Major concerns | No concerns | No concerns | Low |
| Lavender:Lippia citriodora | 0 | Major concerns | Low risk | No concerns | Major concerns | No concerns | No concerns | Very low |
| Lavender:Mint | 0 | Some concerns | Low risk | No concerns | Major concerns | No concerns | No concerns | Low |
| Lemon:Lippia alba | 0 | Some concerns | Low risk | No concerns | Major concerns | No concerns | No concerns | Low |
| Lemon:Lippia citriodora | 0 | Some concerns | Low risk | No concerns | Major concerns | No concerns | No concerns | Low |
| Lemon:Mint | 0 | Some concerns | Low risk | No concerns | Major concerns | No concerns | No concerns | Low |
| Lippia alba:Mint | 0 | Some concerns | Low risk | No concerns | Major concerns | No concerns | No concerns | Low |
| Lippia citriodora:Mint | 0 | Some concerns | Low risk | No concerns | Major concerns | No concerns | No concerns | Low |

**Table S3.2 Efficacy by TAI measure for anxiety.**

| **Comparison** | **Number of studies** | **Within-study bias** | **Reporting bias** | **Indirectness** | **Imprecision** | **Heterogeneity** | **Incoherence** | **Confidence rating** |
| --- | --- | --- | --- | --- | --- | --- | --- | --- |
| Alba:Citriodora | 1 | Major concerns | Low risk | No concerns | Major concerns | No concerns | No concerns | Very low |
| Alba:Control | 2 | Major concerns | Low risk | No concerns | Some concerns | Some concerns | No concerns | Very low |
| Citriodora:Control | 1 | Major concerns | Low risk | No concerns | Major concerns | No concerns | No concerns | Very low |
| Citrus:Control | 2 | Some concerns | Low risk | No concerns | No concerns | Some concerns | Major concerns | Very low |
| Citrus:Lavender | 1 | Some concerns | Low risk | No concerns | Some concerns | Some concerns | No concerns | Low |
| Control:Copaiba | 1 | Major concerns | Low risk | No concerns | Major concerns | No concerns | No concerns | Very low |
| Control:Lavender | 7 | Some concerns | Low risk | No concerns | No concerns | Major concerns | No concerns | Low |
| Control:Lemon | 1 | Some concerns | Low risk | No concerns | Some concerns | Some concerns | No concerns | Low |
| Control:Rose | 3 | Some concerns | Low risk | No concerns | Major concerns | No concerns | No concerns | Low |
| Lavender:Rose | 1 | Some concerns | Low risk | No concerns | Major concerns | No concerns | No concerns | Low |
| Alba:Citrus | 0 | Some concerns | Low risk | No concerns | Major concerns | No concerns | No concerns | Low |
| Alba:Copaiba | 0 | Major concerns | Low risk | No concerns | Major concerns | No concerns | No concerns | Very low |
| Alba:Lavender | 0 | Some concerns | Low risk | No concerns | Major concerns | No concerns | No concerns | Low |
| Alba:Lemon | 0 | Some concerns | Low risk | No concerns | Major concerns | No concerns | No concerns | Low |
| Alba:Rose | 0 | Some concerns | Low risk | No concerns | Major concerns | No concerns | No concerns | Low |
| Citriodora:Citrus | 0 | Some concerns | Low risk | No concerns | Major concerns | No concerns | No concerns | Low |
| Citriodora:Copaiba | 0 | Major concerns | Low risk | No concerns | Major concerns | No concerns | No concerns | Very low |
| Citriodora:Lavender | 0 | Some concerns | Low risk | No concerns | Major concerns | No concerns | No concerns | Low |
| Citriodora:Lemon | 0 | Some concerns | Low risk | No concerns | Major concerns | No concerns | No concerns | Low |
| Citriodora:Rose | 0 | Major concerns | Low risk | No concerns | Major concerns | No concerns | No concerns | Very low |
| Citrus:Copaiba | 0 | Some concerns | Low risk | No concerns | No concerns | Major concerns | No concerns | Low |
| Citrus:Lemon | 0 | Some concerns | Low risk | No concerns | Major concerns | No concerns | No concerns | Low |
| Citrus:Rose | 0 | Some concerns | Low risk | No concerns | No concerns | Major concerns | No concerns | Low |
| Copaiba:Lavender | 0 | Some concerns | Low risk | No concerns | Major concerns | No concerns | No concerns | Low |
| Copaiba:Lemon | 0 | Major concerns | Low risk | No concerns | Major concerns | No concerns | No concerns | Very low |
| Copaiba:Rose | 0 | Major concerns | Low risk | No concerns | Major concerns | No concerns | No concerns | Very low |
| Lavender:Lemon | 0 | Some concerns | Low risk | No concerns | Major concerns | No concerns | No concerns | Low |
| Lemon:Rose | 0 | Some concerns | Low risk | No concerns | Major concerns | No concerns | No concerns | Low |


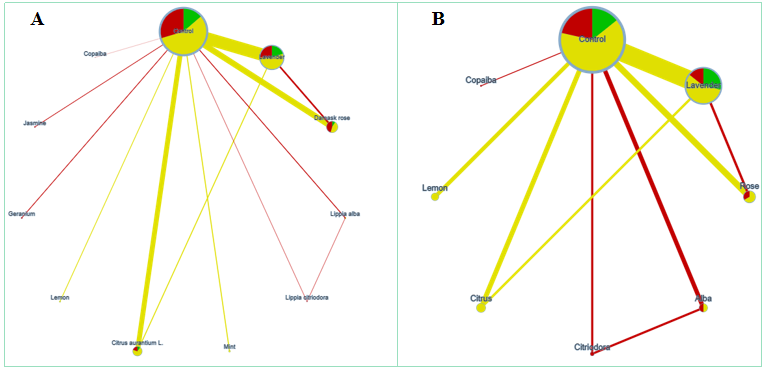


**Figure S3.1** Efficacy by SAI (A) and TAI (B) measure for anxiety. Node size by: sample size; Node color by: risk of bias; Edge width by: number of studies; Edge color by: average of RoB.


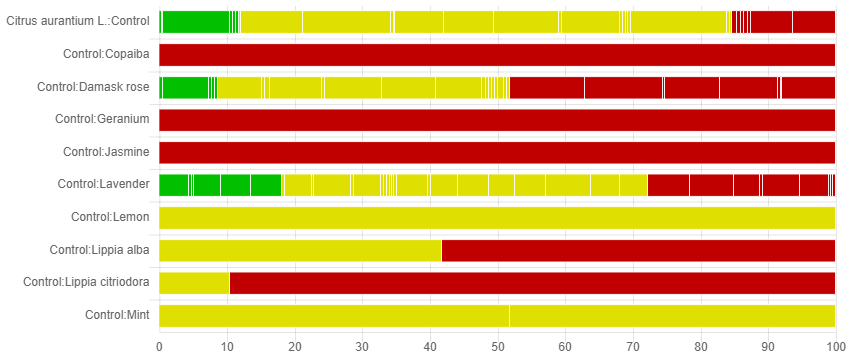


**A**


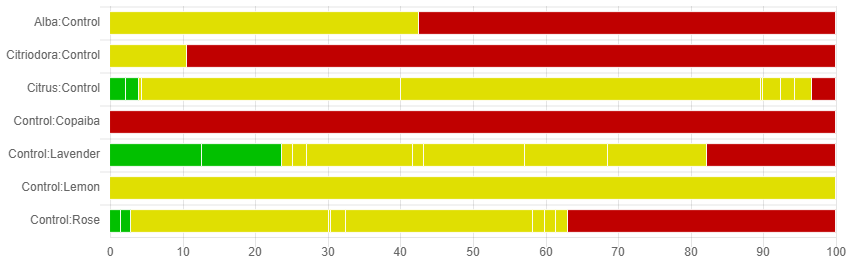


**B**

**Figure S3.2** Efficacy by SAI (A) and TAI (B) measure for anxiety. The bar chart shows the contributions of each piece of study to the network estimate.

***Appendix 4*** ***Network plot - All trials***

###
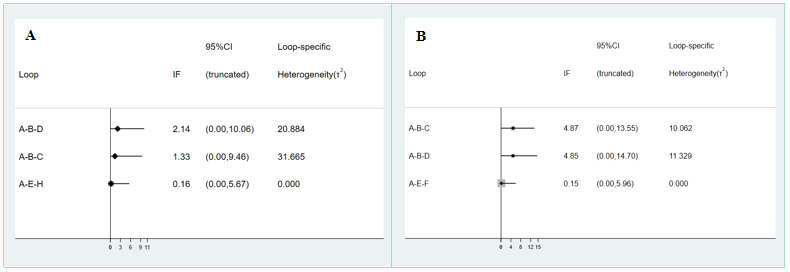


### Figure S4 Inconsistency test for closed loops. (A) SAI, A: Control, B: Lavender, C: Damask rose, D: Citrus aurantium L., E: Lippia alba, F: Lippia citriodora; (B) TAI, A: Control, B: Lavender, C: Citrus aurantium L., D: Lippia alba, E: Lippia citriodora.

D.

**Table S4 The ranking probabilities for each type of essential oil(_%)**

| rank | Control | Lavender | Damask rose | Citrus aurantium L. | Lippia alba | Mint | Lemon | Lippia citriodora | Geranium | Jasmine | Copaiba |
| --- | --- | --- | --- | --- | --- | --- | --- | --- | --- | --- | --- |
| 1 | 0 | 0 | 0.5 | 8.7 | 3.1 | 4.1 | 15.6 | 4.8 | 3.2 | 59.1 | 0.7 |
| 2 | 0 | 0.2 | 4 | 30 | 8.4 | 9.6 | 18.2 | 7.9 | 5 | 15 | 1.7 |
| 3 | 0 | 1.8 | 10.9 | 29.8 | 10.5 | 11.2 | 11.8 | 8.5 | 5.4 | 8 | 2.2 |
| 4 | 0 | 7.3 | 20 | 17.1 | 11.2 | 12.3 | 10.1 | 8.6 | 5.7 | 5.1 | 2.6 |
| 5 | 0 | 16.5 | 23.3 | 8.5 | 12.1 | 10.7 | 8.1 | 8.1 | 5.8 | 3.8 | 3.1 |
| 6 | 0.1 | 24 | 18.7 | 4 | 12.4 | 11.4 | 7.5 | 8.4 | 6.9 | 2.8 | 4 |
| 7 | 0.8 | 25.1 | 13.1 | 1.4 | 13.5 | 11.3 | 8.2 | 10.7 | 8.3 | 2.2 | 5.5 |
| 8 | 6.4 | 17.6 | 6.9 | 0.3 | 12.8 | 12.5 | 7.9 | 13.1 | 11.4 | 1.9 | 9.1 |
| 9 | 24.2 | 6.9 | 2.2 | 0.2 | 10 | 9.7 | 6.6 | 12 | 13.8 | 1.1 | 13.3 |
| 10 | 44.5 | 0.7 | 0.4 | 0 | 4.4 | 5 | 3.5 | 9.4 | 14.3 | 0.6 | 17.3 |
| 11 | 24.1 | 0 | 0 | 0 | 1.7 | 2.3 | 2.5 | 8.6 | 20.1 | 0.3 | 40.5 |

***Appendix 5 Results of subgroup analysis for various outcomes.***

1. **Table S5 Meta-analyses of essential oils on anxiety and physiological parameters.**

| **Variable** | **NO.** | **WMD (95%CI)** | **I^2^ (%)** | **P heterogeneity** | **P within group** |
| --- | --- | --- | --- | --- | --- |
| **Subgroups analyses of EOs on SAI.** | | | | | |
| Overall effect | 50 | -6.628 (-8.173, -5.084) | 93.2 | < 0.001 | < 0.001 |
| **Type of EOs** | | | | | |
| Lavender | 20 | -5.346 (-7.484, -3.209) | 93.0 | < 0.001 | < 0.001 |
| Damask rose | 11 | -7.008 (-11.510, -2.506) | 96.2 | < 0.001 | 0.002 |
| Citrus aurantium L. | 10 | -9.880 (-13.01, -6.75) | 78.1 | < 0.001 | < 0.001 |
| Lippia alba | 2 | -6.039 (-8.339, -3.740) | 0 | 0.946 | < 0.001 |
| Mint | 2 | -6.000 (-8.339, -3.740) | 64.3 | 0.094 | 0.009 |
| Lemon | 1 | -8.240 (-10.16, -6.32) | - | - | < 0.001 |
| Lippia citriodora | 1 | -4.940 (-8.181, -1.699) | - | - | 0.003 |
| Geranium | 1 | -2.880 (-6.396, 0.636) | - | - | 0.108 |
| Jasmine | 1 | -13.59 (-16.602, -10.578) | - | - | < 0.001 |
| Copaiba | 1 | 0.200 (-2.090, 2.490) | - | - | 0.864 |
| **Country** | | | | | |
| Iran | 33 | -7.665 (-9.697, -5.633) | 93.2 | < 0.001 | < 0.001 |
| Perú | 3 | -5.671 (-7.547, -3.796) | 0 | 0.861 | < 0.001 |
| Turkey | 8 | -4.558 (-8.455, -0.661) | 96.1 | < 0.001 | 0.022 |
| China | 3 | -0.033 (-1.512, 1.445) | 0 | 0.538 | 0.965 |
| Brazil | 1 | -11 (-19.861, -2.139) | - | - | 0.015 |
| Philippines | 1 | -12.4 (-20.977, -3.823) | - | - | 0.005 |
| Singapore | 1 | -3.7 (-8.465, 1.065) | - | - | 0.128 |
| **Causes of anxiety** | | | | | |
| Spontaneous anxiety | 4 | -4.069 (-7.445, -0.694) | 80.5 | 0.001 | 0.018 |
| Delivery-related anxiety | 6 | -6.148 (-12.189, -0.108) | 95.9 | < 0.001 | 0.046 |
| Operation-related anxiety | 14 | -8.300 (-11.090, -5.510) | 85.1 | < 0.001 | < 0.001 |
| Cardiovascular disease-related anxiety | 5 | -8.301 (-11.020, -5.582) | 74.1 | 0.004 | < 0.001 |
| Renal disease-related anxiety | 1 | 2.300 (1.013, 3.587) | - | - | < 0.001 |
| Anxiety caused by fractures | 1 | -19.96 (-26.963, -12.957) | - | - | < 0.001 |
| Invasive examinations induced anxiety | 12 | -4.572 (-7.133, -2.010) | 91.8 | < 0.001 | < 0.001 |
| Menopause-related anxiety | 1 | -4.480 (-7.306, -1.654) |  |  |  |
| Puncture-related anxiety | 3 | -7.777 (-10.053, -5.500) | 0 | 0.420 | 0.002 |
| Malignancy-related anxiety | 2 | -4.176 (-15.779, 7.427) | 85.5 | 0.009 | < 0.001 |
| Burn-related anxiety | 1 | -15.13 (-20.354, -9.906) | - | - | 0.481 |
| **Cumulative duration of intervention** | | | | | |
| ≤ 10 min | 7 | -6.973 (-10.415, -3.531) | 79.4 | < 0.001 | < 0.001 |
| 10 < t ≤ 30 min | 32 | -5.815 (-7.779, -3.851) | 93.3 | < 0.001 | < 0.001 |
| 30 < t ≤ 100 min | 2 | -11.334 (-16.237, -6.431) | 71.0 | 0.063 | < 0.001 |
| 100 < t ≤ 500 min | 6 | -7.708 (-12.836, -2.579) | 94.9 | < 0.001 | 0.003 |
| > 500 min | 3 | -9.205 (-14.203, -4.207) | 87.5 | < 0.001 | < 0.001 |
| **Subgroups analyses of EOs on TAI.** | | | | | |
| Overall effect | 17 | -4.967 (-6.730, -3.204) | 93.9 | < 0.001 | < 0.001 |
| **Type of EOs** | | | | | |
| Lavender | 7 | -5.484 (-8.160, -2.807) | 96.1 | < 0.001 | < 0.001 |
| Damask rose | 3 | -1.712 (-5.946, 2.521) | 86.3 | 0.001 | 0.428 |
| Citrus aurantium L. | 2 | -10.93 (-13.931, -7.929) | 0 | 1.000 | < 0.001 |
| Lippia alba | 2 | -4.392 (-6.826, -1.958) | 0 | 0.952 | < 0.001 |
| Lippia citriodora | 1 | -3.510 (-6.711, -0.309) | - | - | 0.032 |
| Lemon | 1 | -8.490 (-9.723, -7.257) | - | - | < 0.001 |
| Copaiba | 1 | 0.200 (-2.090, 2.490) | - | - | 0.864 |
| **Country** | | | | | |
| Iran | 11 | -5.560 (-8.293, -2.827) | 92.1 | < 0.001 | < 0.001 |
| Perú | 3 | -4.069 (-6.006, -2.132) | 0 | 0.910 | < 0.001 |
| Turkey | 2 | -6.055 (-10.423, -1.687) | 98.9 | < 0.001 | 0.007 |
| China | 1 | 0.200 (-2.090, 2.490) | - | - | 0.864 |
| **Causes of anxiety** | | | | | |
| Spontaneous anxiety | 4 | -2.794 (-5.281, -0.307) | 62.4 | 0.047 | 0.028 |
| Delivery-related anxiety | 2 | -0.521 (-2.181, 1.139) | 0 | 0.891 | 0.539 |
| Operation-related anxiety | 5 | -8.947 (-11.061, -6.833) | 34.3 | 0.192 | < 0.001 |
| Cardiovascular disease-related anxiety | 1 | -8.490 (-9.723, -7.257) | - | - | < 0.001 |
| Invasive examinations-related anxiety | 2 | -3.250 (-12.315, 5.815) | 96.4 | < 0.001 | 0.482 |
| Menopause-related anxiety | 1 | -0.650 (-2.849, 1.549) | - | - | 0.562 |
| Puncture-related anxiety | 1 | -8.274 (-8.797, -7.751) | - | - | < 0.001 |
| Malignancy-related anxiety | 1 | -3.817 (-4.593, -3.041) | - | - | < 0.001 |
| **Cumulative duration of intervention** | | | | | |
| ≤ 10 min | 2 | -7.090 (-8.922, -5.259) | 7.2 | 0.299 | < 0.001 |
| 10 < t ≤ 30 min | 11 | -4.935 (-7.882, -1.989) | 94.3 | < 0.001 | 0.001 |
| 100 < t ≤ 500 min | 2 | -3.848 (-4.606, -3.089) | 0 | 0.722 | < 0.001 |
| > 500 min | 2 | -4.625 (-12.307, 3.057) | 97.3 | < 0.001 | 0.238 |
| **Subgroups analyses of EOs on SBP.** | | | | | |
| Overall effect | 12 | -6.825 (-10.530, -3.121) | 82.6 | < 0.001 | < 0.001 |
| **Type of EOs** | | | | | |
| Lavender | 5 | -3.952 (-7.437, -0.466) | 52.1 | 0.080 | 0.026 |
| Lemon | 1 | -33.20 (-42.252, -24.148) | - | - | < 0.001 |
| Citrus aurantium L. | 4 | -7.951 (-9.971, -5.931) | 0 | < 0.001 | < 0.001 |
| Mint | 1 | -0.660 (-8.673, 7.353) | - | - | 0.872 |
| Geranium | 1 | 0.000 (-7.151, 7.151) | - | - | 1 |
| **Country** |  |  |  |  |  |
| Iran | 6 | -9.047 (-16.013, -2.081) | 87.4 | < 0.001 | 0.011 |
| Turkey | 3 | -3.527 (-8.479, 1.425) | 60.1 | 0.081 | 0.163 |
| China | 1 | -4.050 (-19.770, 11.670) | - | - | 0.614 |
| Brazil | 1 | -7.900 (-10.389, -5.411) | - | - | < 0.001 |
| Singapore | 1 | -2.880 (-11.724, 5.964) | - | - | 0.523 |
| **Causes of anxiety** | | | | | |
| Delivery-related anxiety | 1 | 0.000 (-7.151, 7.151) | - | - | 1 |
| Operation-related anxiety | 3 | -4.256 (-9.159, 0.648) | 0 | 0.929 | 0.089 |
| Cardiovascular disease-related anxiety | 2 | -16.859 (-48.747, 15.029) | 96.4 | < 0.001 | 0.300 |
| Invasive examinations-related anxiety | 5 | -5.877 (-10.129, -1.626) | 76.3 | 0.002 | 0.007 |
| Menopause-related anxiety | 1 | -6.825 (-10.530, -3.121) | - | - | < 0.001 |
| **Cumulative duration of intervention.** | | | | | |
| ≤ 10 min | 2 | -4.867 (-10.759, 1.026) | 0 | 0.913 | 0.106 |
| 10 < t ≤ 30 min | 8 | -5.372 (-8.484, -2.259) | 72.6 | 0.001 | 0.001 |
| 100 < t ≤ 500 min | 1 | -0.660 (-8.673, 7.353) | - | - | 0.872 |
| > 500 min | 1 | -33.20 (-42.252, -24.148) | - | - | < 0.001 |
| **Subgroups analyses of EOs on DBP.** | | | | | |
| Overall effect | 12 | -2.114 (-4.354, 0.126) | 82.6 | < 0.001 | 0.064 |
| **Type of EOs** | | | | | |
| Lavender | 5 | -1.627 (-4.655, 1.401) | 73.6 | 0.004 | 0.292 |
| Lemon | 1 | 1.740 (-2.018, 5.498) | - | - | 0.364 |
| Citrus aurantium L. | 4 | -4.036 (-7.037, -1.035) | 69.9 | 0.019 | 0.008 |
| Mint | 1 | 0.530 (-2.513, 3.573) | - | - | 0.733 |
| Geranium | 1 | -5.000 (-10.614, 0.614) | - | - | 0.081 |
| **Country** | | | | | |
| Iran | 6 | -1.283 (-3.532, 0.965) | 58.5 | 0.034 | 0.263 |
| Turkey | 3 | -2.884 (-8.439, 2.671) | 86.7 | 0.001 | 0.309 |
| China | 1 | -1.510 (-10.876, 7.856) | - | - | 0.752 |
| Brazil | 1 | -6.400 (-7.886, -4.914) | - | - | < 0.001 |
| Singapore | 1 | 0.200 (-4.617, 5.017) | - | - | 0.935 |
| **Causes of anxiety** | | | | | |
| Delivery-related anxiety | 1 | -5.000 (-10.614, 0.614) | - | - | 0.081 |
| Operation-related anxiety | 3 | -0.072 (-2.977, 2.832) | 0 | 0.949 | 0.961 |
| Cardiovascular disease-related anxiety | 2 | 1.009 (-1.356, 3.374) | 0 | 0.624 | 0.403 |
| Invasive examinations-related anxiety | 5 | -2.745 (-5.952, 0.462) | 81.7 | < 0.001 | 0.093 |
| Menopause-related anxiety | 1 | -6.400 (-7.886, -4.914) | - | - | < 0.001 |
| **Cumulative duration of intervention** | | | | | |
| ≤ 10 min | 2 | -0.228 (-3.868, 3.412) | 0 | 0.771 | 0.902 |
| 10 < t ≤ 30 min | 8 | -3.235 (-6.002, -0.467) | 85.9 | < 0.001 | 0.022 |
| 100 < t ≤ 500 min | 1 | 0.530 (-2.513, 3.573) | - | - | 0.733 |
| > 500 min | 1 | 1.740 (-2.018, 5.498) | - | - | 0.364 |
| **Subgroups analyses of EOs on HR.** | | | | | |
| Overall effect | 12 | -3.433 (-5.505, -1.361) | 82.4 | < 0.001 | 0.001 |
| **Type of EOs** | | | | | |
| Lavender | 5 | -3.630 (-6.713, -0.547) | 66.0 | 0.019 | 0.021 |
| Lemon | 1 | -13.710 (-18.307, -9.113) | - | - | < 0.001 |
| Citrus aurantium L. | 4 | -2.999 (-4.422, -1.576) | 15.7 | 0.313 | < 0.001 |
| Mint | 1 | 0.290 (-0.969, 1.549) | - | - | 0.652 |
| Geranium | 1 | 2.000 (-2.605, 6.605) | - | - | 0.395 |
| **Country** | | | | | |
| Iran | 6 | -4.199 (-7.779, -0.619) | 90.5 | < 0.001 | 0.022 |
| Turkey | 3 | -3.498 (-9.246, 2.251) | 79.2 | 0.008 | 0.233 |
| China | 1 | -0.150 (-9.996, 9.696) | - | - | 0.976 |
| Brazil | 1 | -2.200 (-3.595, -0.805) | - | - | 0.002 |
| Singapore | 1 | -2.300 (-7.664, 3.064) | - | - | 0.401 |
| **Causes of anxiety** | | | | | |
| Delivery-related anxiety | 1 | 2.000 (-2.605, 6.605) | - | - | 0.395 |
| Operation-related anxiety | 3 | -1.887 (-5.508, 1.735) | 0 | 0.931 | 0.307 |
| Cardiovascular disease-related anxiety | 2 | -6.528 (-20.243, 7.187) | 97.0 | < 0.001 | 0.351 |
| Invasive examinations-related anxiety | 5 | -4.408 (-6.890, -1.926) | 65.1 | 0.022 | < 0.001 |
| Menopause-related anxiety | 1 | -2.200 (-3.595, -0.805) | - | - | 0.002 |
| **Cumulative duration of intervention** | | | | | |
| ≤ 10 min | 2 | -1.540 (-6.448, 3.368) | 0 | 0.750 | 0.538 |
| 10 < t ≤ 30 min | 8 | -3.184 (-5.050, -1.318) | 65.6 | 0.005 | 0.001 |
| 100 < t ≤ 500 min | 1 | 0.290 (-0.969, 1.549) | - | - | 0.652 |
| > 500 min | 1 | -13.710 (-18.307, -9.113) | - | - | < 0.001 |
| **Subgroups analyses of EOs on RR.** | | | | | |
| Overall effect | 10 | -0.533 (-1.524, 0.458) | 97.2 | < 0.001 | 0.292 |
| **Type of EOs** | | | | | |
| Lavender | 4 | 0.039 (-0.493, 0.570) | 25.5 | 0.259 | 0.887 |
| Citrus aurantium L. | 4 | -1.402 (-3.066, 0.263) | 0 | 95.4 | 0.099 |
| Mint | 1 | 0.210 (0.017, 0.403) | - | - | 0.033 |
| Geranium | 1 | 0.000 (-1.592, 1.592) | - | - | 1 |
| **Country** | | | | | |
| Iran | 5 | -0.252 (-2.283, 1.779) | 97.4 | < 0.001 | 0.808 |
| Turkey | 2 | 0.000 (-0.306, 0.306) | 0 | 1 | 1 |
| China | 1 | -0.510 (-2.005, 0.985) | - | - | 0.504 |
| Brazil | 1 | -2.300 (-2.605, -1.995) | - | - | < 0.001 |
| Singapore | 1 | -0.900 (-2.354, 0.554) | - | - | 0.225 |
| **Causes of anxiety** | | | | | |
| Delivery-related anxiety | 1 | 0.000 (-1.592, 1.592) | - | - | 1 |
| Operation-related anxiety | 3 | -0.434 (-1.249, 0.381) | 0 | 0.661 | 0.296 |
| Cardiovascular disease-related anxiety | 1 | 0.210 (0.017, 0.403) | - | - | 0.033 |
| Invasive examinations-related anxiety | 4 | -0.362 (-2.698, 1.974) | 0 | 97.7 | 0.761 |
| Menopause-related anxiety | 1 | -2.300 (-2.605, -1.995) | - | - | < 0.001 |
| **Cumulative duration of intervention** | | | | | |
| ≤ 10 min | 2 | -0.221 (-1.205, 0.763) | 0 | 0.615 | 0.660 |
| 10 < t ≤ 30 min | 7 | 0.706 (-2.061, 0.649) | 97.0 | < 0.001 | 0.307 |
| 100 < t ≤ 500 min | 1 | 0.210 (0.017, 0.403) | - | - | 0.033 |

1. **Figure S5. The Forest plots for subgroup analyses.**

A.

**
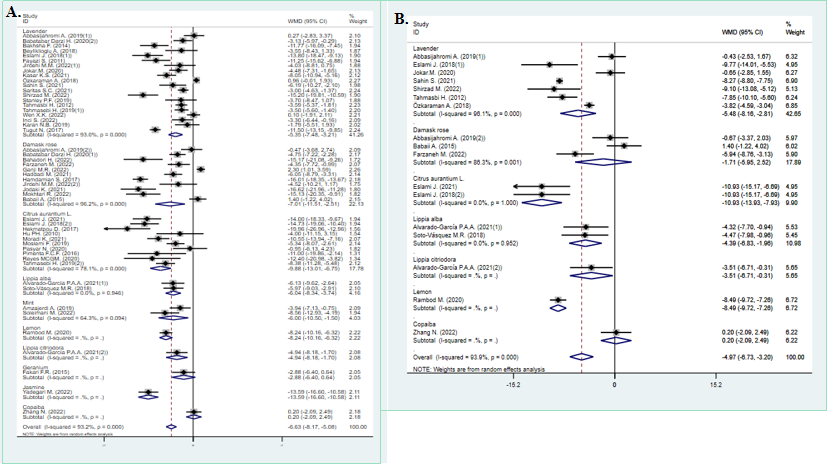
**

F**igure S5.1a** Forest plot showing the therapeutic effect of essential oils compared to placebo for types of essential oils. A. state anxiety (SAI scores); B. trait anxiety (TAI scores).


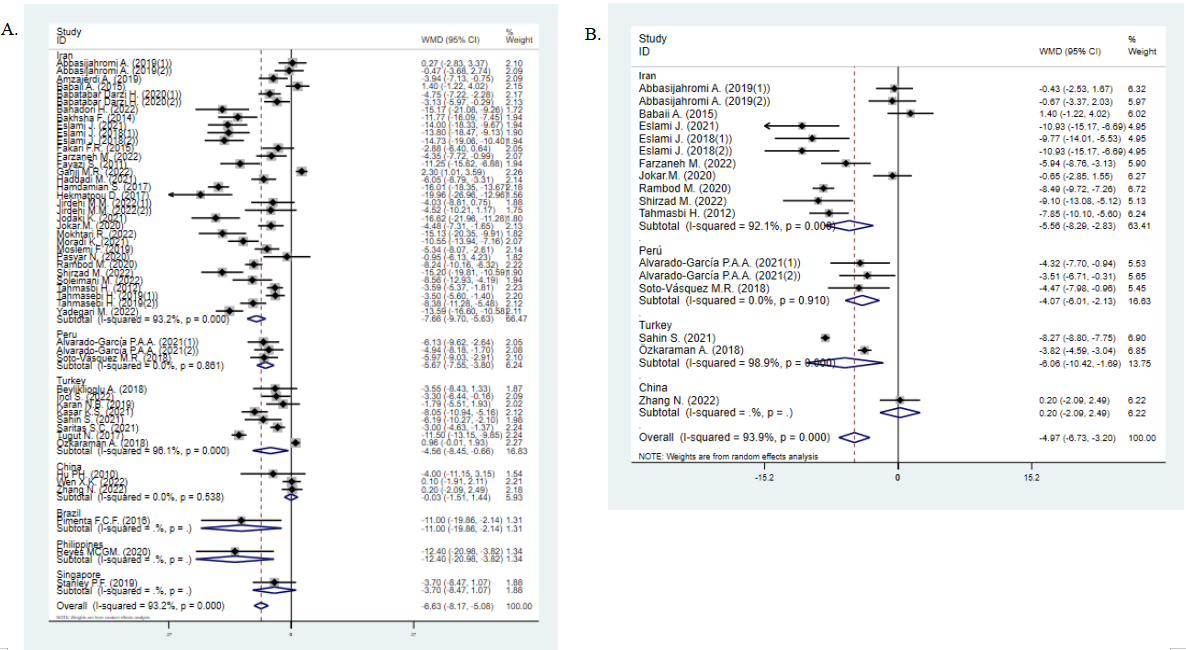


**Figure S5.1b** Forest plot showing the therapeutic effect of essential oils compared to placebo for different countries. A. state anxiety (SAI scores); B. trait anxiety (TAI scores).


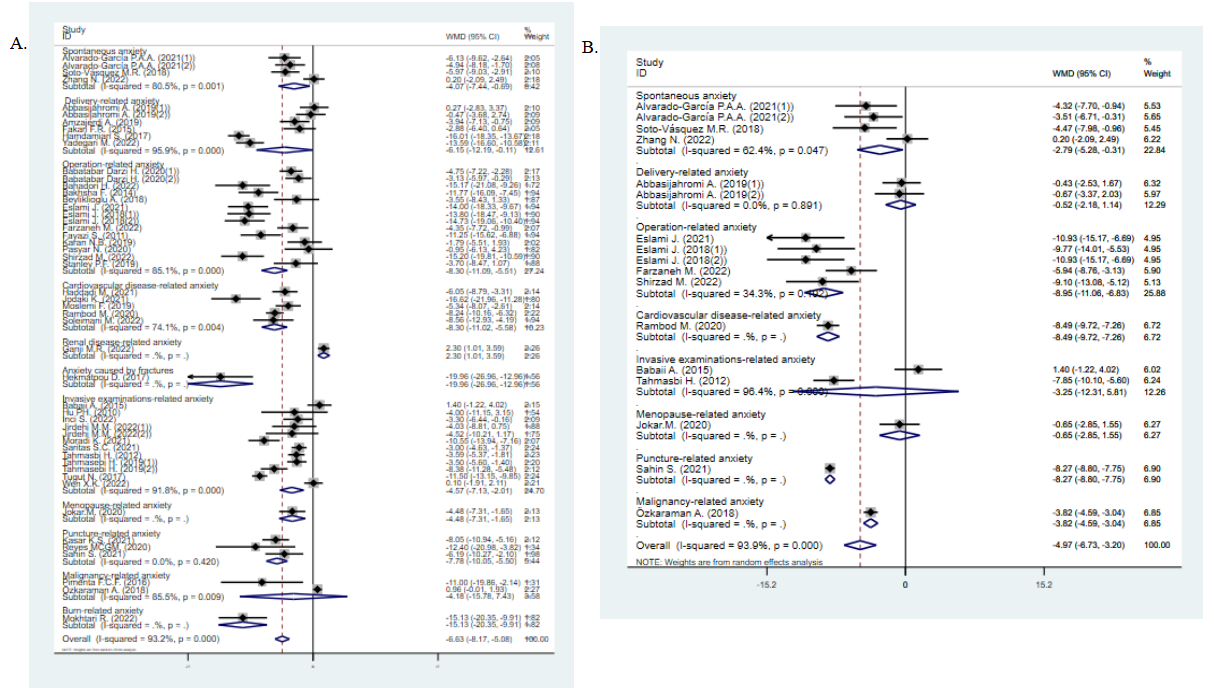


**Figure S5.1c** Forest plot showing the therapeutic effect of essential oils compared to placebo for different causes of anxiety. A. state anxiety (SAI scores); B. trait anxiety (TAI scores).


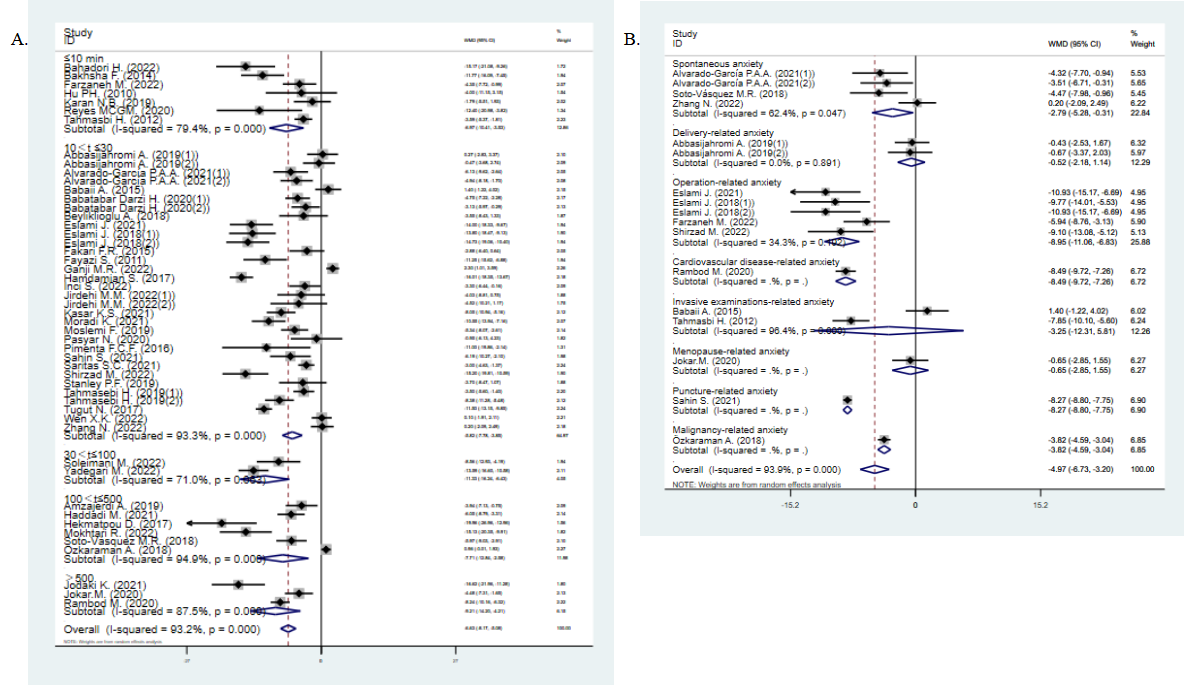
**Figure S5.1d** Forest plot showing the therapeutic effect of essential oils compared to placebo for different intervention duration. A. state anxiety (SAI scores); B. trait anxiety (TAI scores).

**
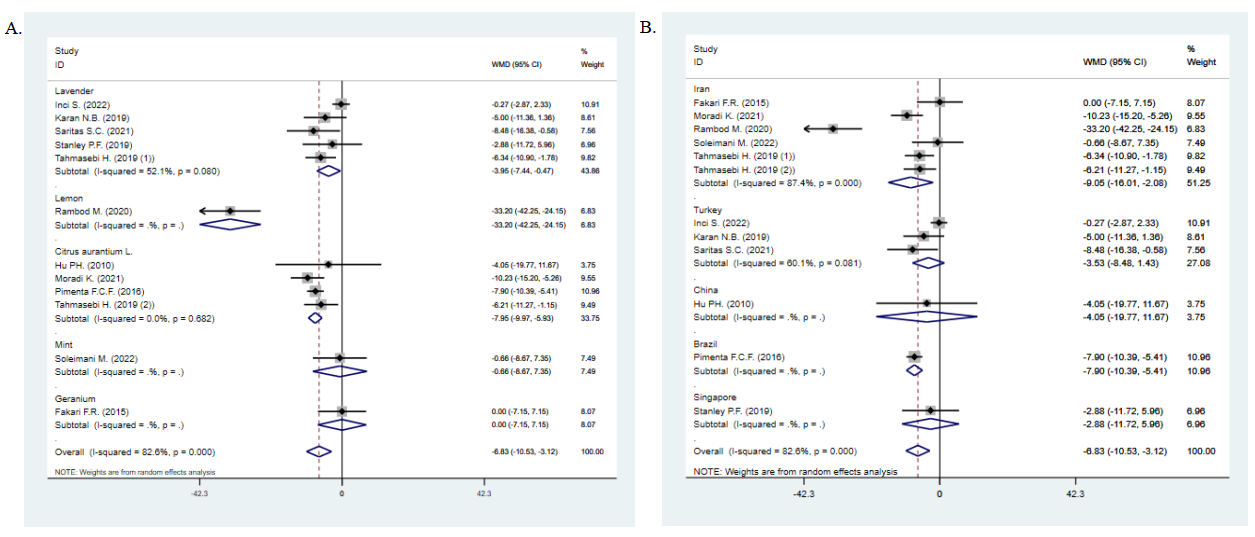
Figure S5.2a** Forest plot showing the effect of different reasons on SBP. A. the effect of different types of essential oils on SBP; B. the effect of different countries on SBP.


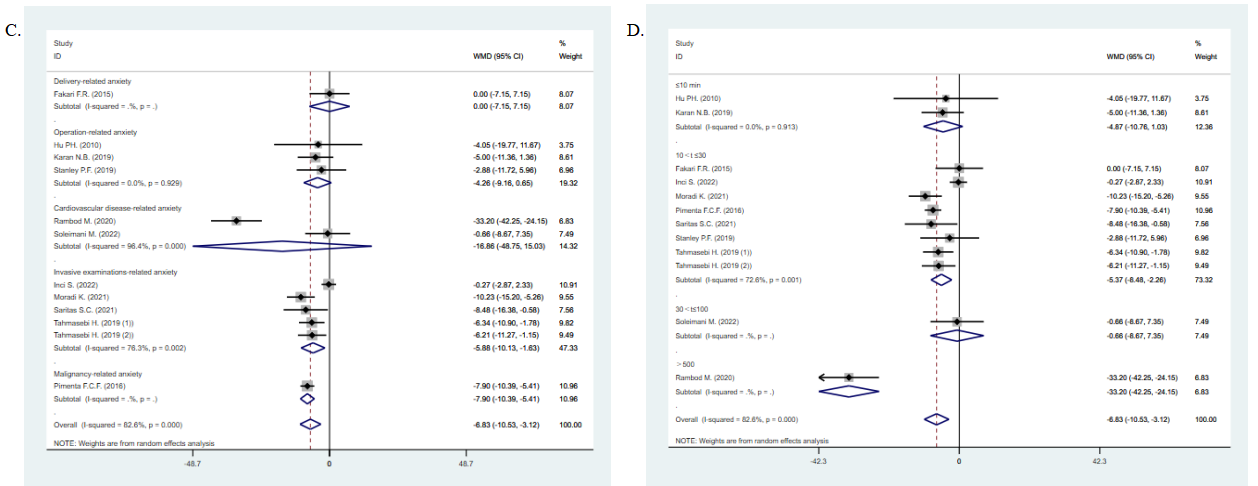


**Figure S5.2a** Forest plot showing the effect of different reasons on SBP. C. the effect of different causes of anxiety on SBP; D. the effect of different intervention duration on SBP.


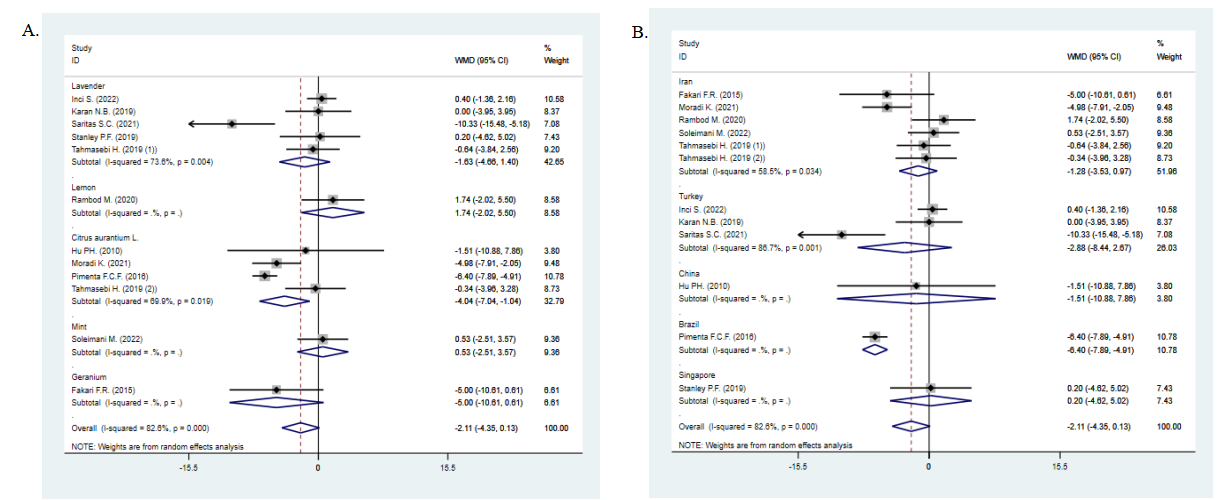


**Figure S5.2b** Forest plot showing the effect of different reasons on DBP. A. the effect of different types of essential oils on DBP; B. the effect of different countries on DBP.


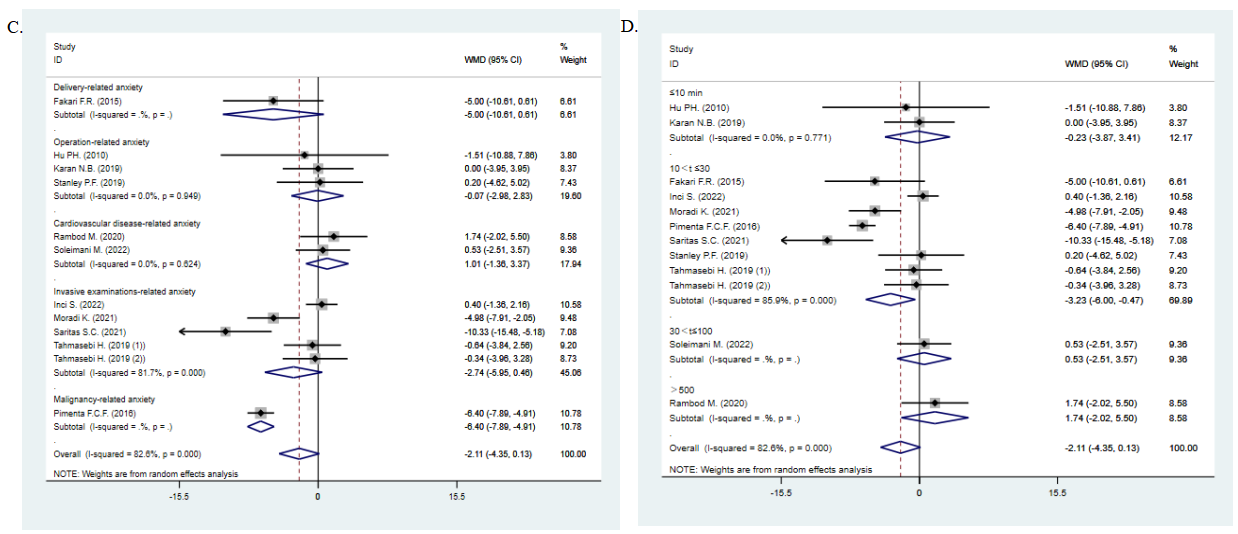


**Figure S5.2b** Forest plot showing the effect of different reasons on DBP. C. the effect of different causes of anxiety on DBP; D.the effect of different intervention duration on DBP.


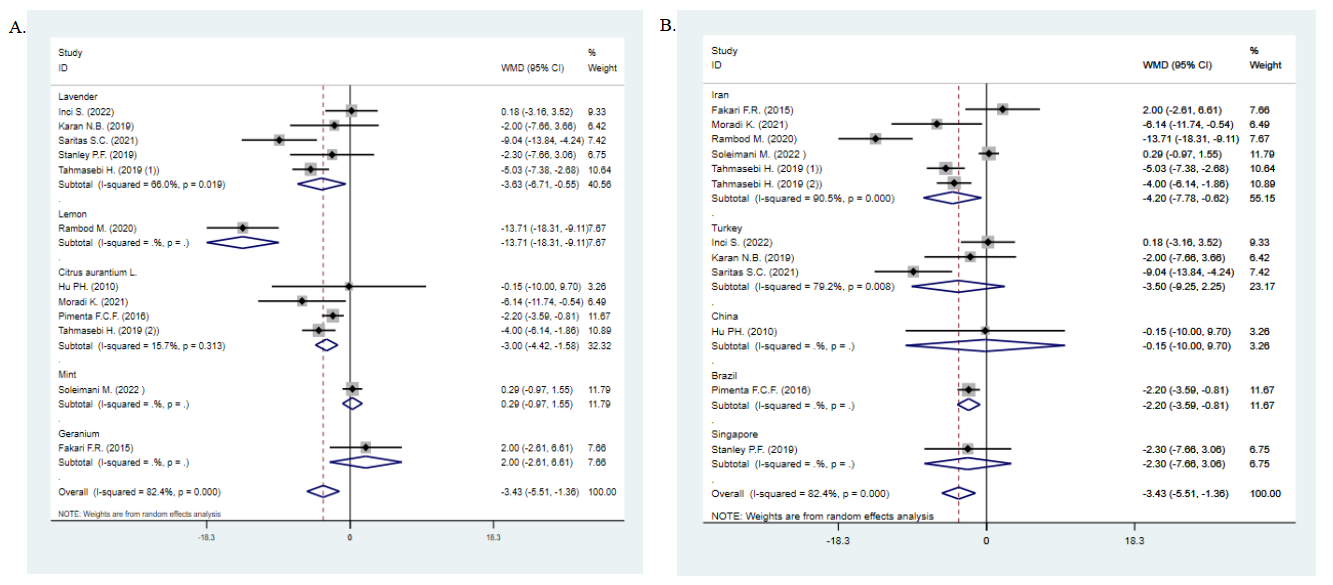


**Figure S5.2c** Forest plot showing the effect of different reasons on HR. A. the effect of different types of essential oils on HR; B. the effect of different countries on HR.


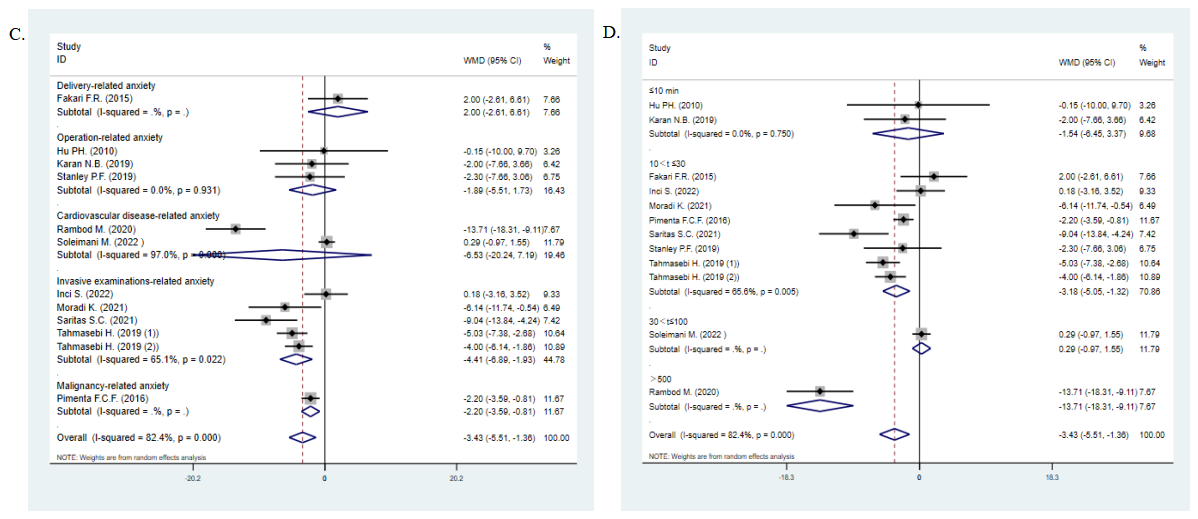


**Figure S5.2c** Forest plot showing the effect of different reasons on HR. C. the effect of different causes of anxiety on HR; D. the effect of different intervention duration on HR.

C.


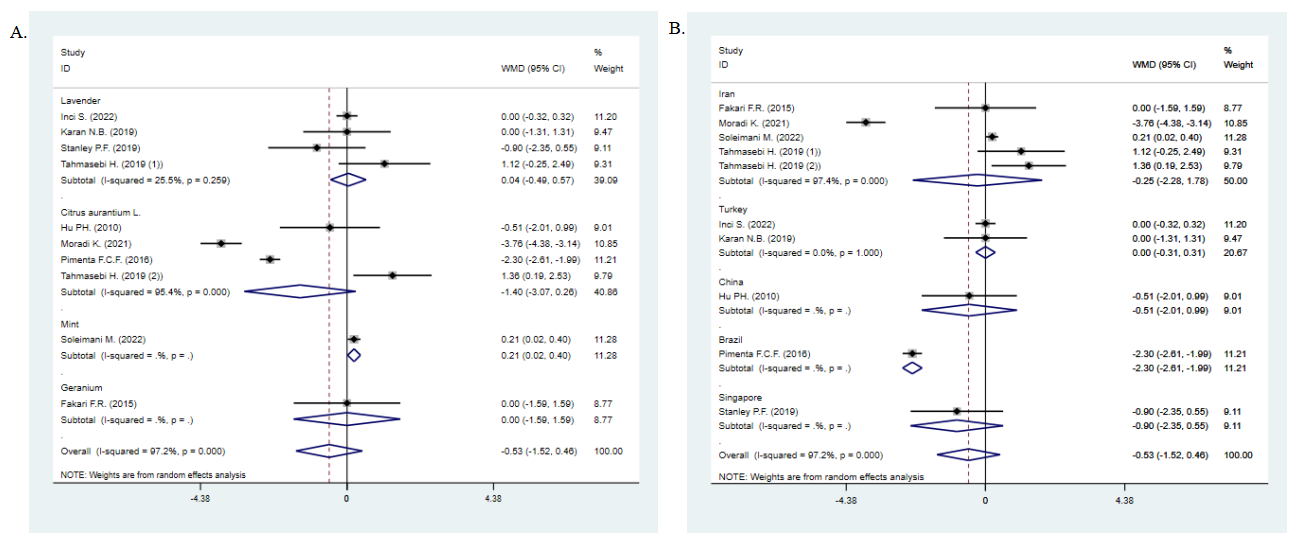


**Figure S5.2d** Forest plot showing the effect of different reasons on RR. A. the effect of different types of essential oils on RR; B. the effect of different countries on RR.


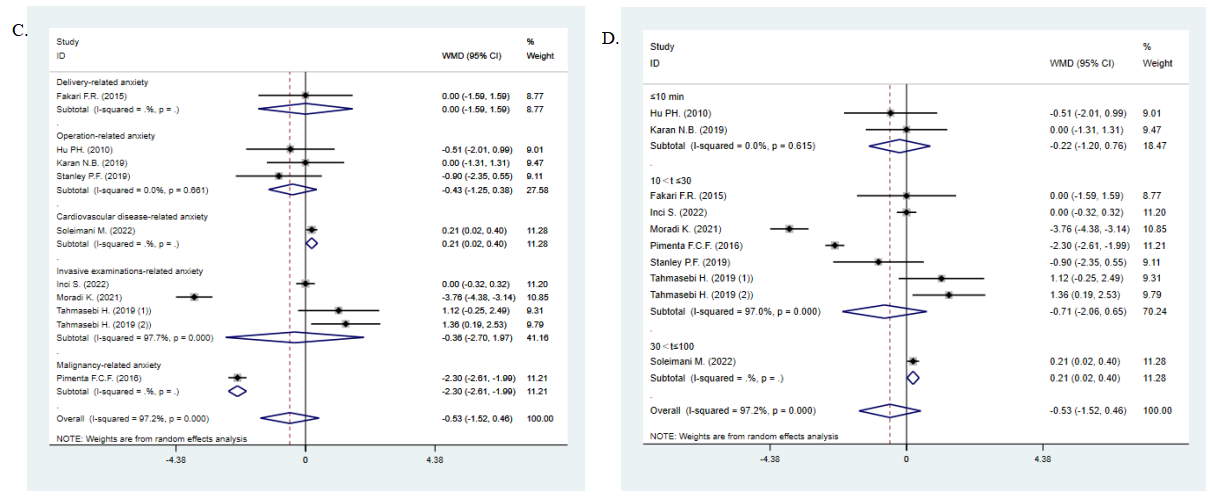


**Figure S5.2d** Forest plot showing the effect of different reasons on RR. C. the effect of different causes of anxiety on RR; D. the effect of different intervention duration on RR.

***Appendix 6 Sensitivity analysis***

***
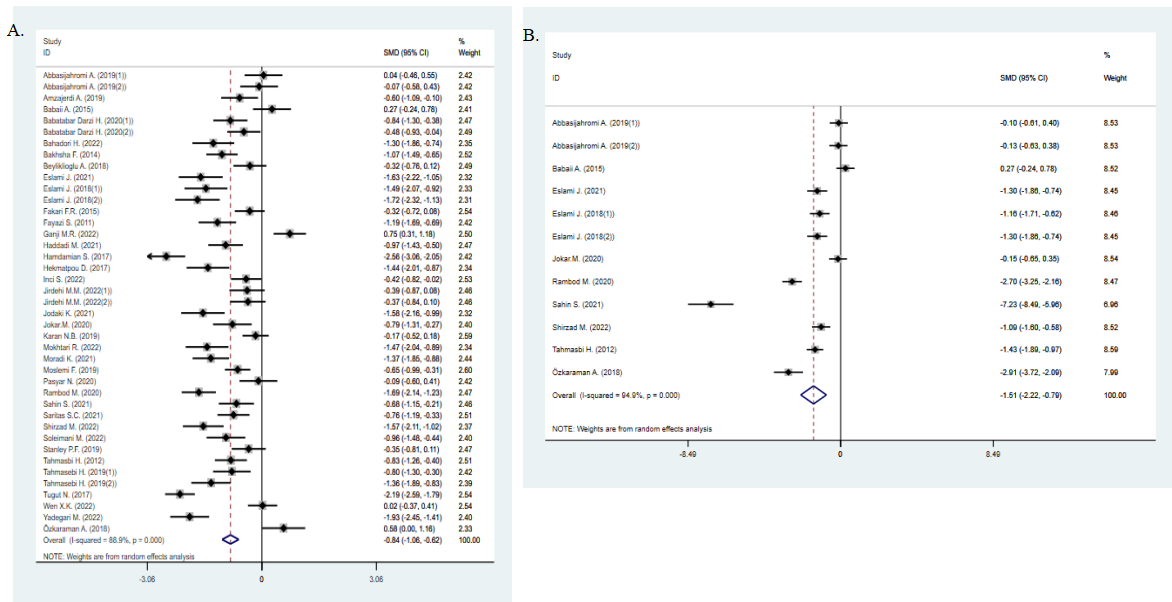
***

**Figure S6** Forest plot showing meta-analysis of the effect of essential oil on anxiety in the experimental group of no less than 30 people. A. state anxiety (SAI scores); B. trait anxiety (TAI scores).


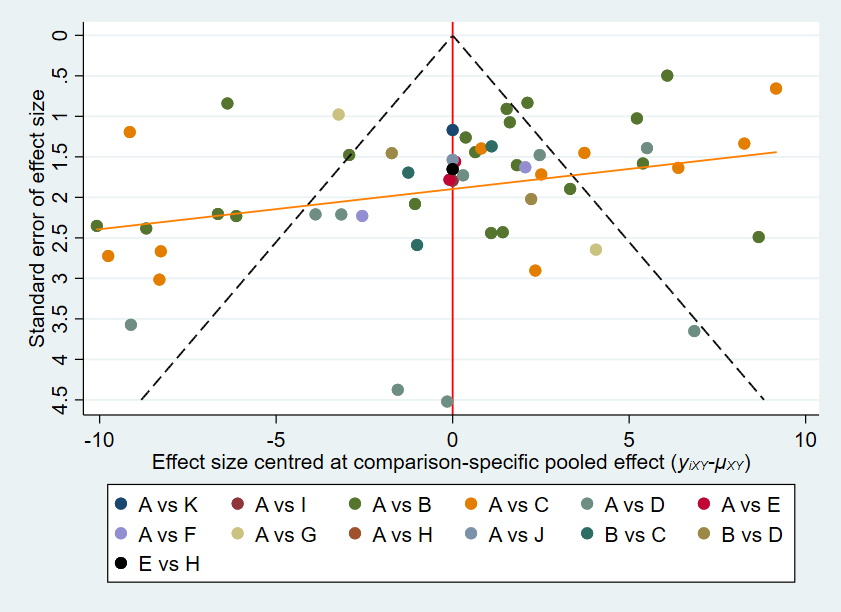
***Appendix 7 Publication Bias***

**Figure S7 Comparison-adjusted funnel plots of anxiety level.**

Intervention: A:Control;B:Lavender;C:Damask rose;D:Citrus aurantium L;E:Lippia abla;F:Mint;G:Lemon;H:Lippia citriodora;I:Geranium;J:Jasmine;K:Copaiba.
